# Disruption of macrophage migration inhibitory factor signaling induces major tumor-associated macrophage phenotypes in human M2 macrophages

**DOI:** 10.1186/s43556-026-00515-3

**Published:** 2026-07-17

**Authors:** Dominik Klaver, Hubert Gander, Beatrice Frena, Michael Martin, Marco Amato, Julia Richter, Renate Pichler, Martin Thurnher

**Affiliations:** 1 Immunotherapy Unit, Department of Urology, Medical University of Innsbruck, Innsbruck, Austria; 2 Avidicure B.V., Oegstgeest, The Netherlands; 3 Central Institute for Blood Transfusion & Department of Immunology (ZIB), Tirol Kliniken GmbH, Innsbruck, Austria; 4 Department of Urology, Medical University of Innsbruck, Innsbruck, Austria

**Keywords:** MIF, p53, NR4A1, ACSL4, CD38, Abemaciclib

## Abstract

Tumor-associated macrophages (TAMs) primarily arise from infiltrating monocytes, yet the mechanisms guiding their differentiation remain unclear. Here, we show that human macrophages rely on autocrine macrophage migration inhibitory factor signaling to suppress p53 during M2-like transition. Disruption of this pathway led to activation of p53 and, unexpectedly, the nuclear receptor NR4A1, inducing a senescence-like state resembling interleukin (IL)-1β⁺ and IL-4 Induced 1 (IL4I1)⁺ TAM subsets observed across multiple cancers. These TAM-like macrophages exhibited a transcriptional program driven by NR4A1, similar to that induced in IL-1β⁺ TAMs by the combined action of tumor necrosis factor α (TNF) and the cyclooxygenase-2-dependent arachidonic acid (AA) metabolite prostaglandin E2. They also upregulated the AA-selective acyl-CoA synthase ACSL4, which promoted cell survival and restrained IL-1β release despite elevated *IL1B* expression. This effect was mediated through induction of the IL4I1⁺ TAM marker CD38, which drove the production of IL-10. Mechanistically, ACSL4 preserved the homeostatic function of stimulator of interferon genes (STING). Loss of ACSL4 resulted in extensive macrophage death and converted STING from a homeostatic regulator into a driver of IL-1 cytokine release. Finally, we show that the CDK4/6 inhibitor abemaciclib repolarized TAM-like macrophages toward a more inflammatory phenotype through off-target inhibition of ACSL4. Abemaciclib enhanced inflammatory signaling by modulating ectodomain shedding, increasing TNF while reducing the release of its natural antagonist, TNF receptor II. Together, these findings clarify mechanisms underlying scRNA-seq–defined TAM phenotypes, identify ACSL4 as a potential therapeutic target, and reveal how abemaciclib can promote inflammatory responses in cancer patients.

**Supplementary Information:**

The online version contains supplementary material available at 10.1186/s43556-026-00515-3.

## Introduction

Macrophages transition from pro-inflammatory M1 to anti-inflammatory M2 states to resolve inflammation [1]. Failure to do so fosters chronic inflammation. Single-cell RNA sequencing (scRNA-seq) has identified tumor-associated macrophage (TAM) subsets with a chronic inflammatory and immunosuppressive phenotype in most human tumors [2–4]. Among these, IL-1ß^+^ TAMs [2, 4] and IL4I1^+^CD274(PD-L1)^+^IDO1^+^ TAMs (IL4I1^+^ TAMs) [3] have emerged as key drivers of pathogenic inflammation and immunosuppression in cancer. TAMs likely arise from monocytes [4]. Although their differentiation is thought to be driven by signals from the tumor microenvironment (TME), the underlying mechanisms remain unclear.

The tumor suppressor and senescence inducer p53 can disrupt the M2 polarization process [5]. Furthermore, p53 can activate cell death pathways [6]. To survive and complete the M2 polarization process, macrophages must therefore prevent undue p53 accumulation. If it nevertheless occurs, potent survival pathways are required to prevent cell death. One way to escape p53-induced cell death is entry into senescence [7], a protective state characterized by limited replication and high secretory activity. Lipid metabolism of senescent cells undergoes significant changes [7]. Fatty acid synthase (FASN), a key enzyme in lipogenesis, is required for senescence induction [8]. Accordingly, fatty acids (FAs) often accumulate in senescent cells. While not a primary trigger, the long-chain polyunsaturated FA (PUFA) arachidonic acid (AA, 20:4n-6) can contribute to senescence. In order to reduce the levels of free (unesterified) AA, cells generally, and senescent cells in particular, activate *PTGS2*, encoding cyclooxygenase-2 (COX-2), to metabolize AA towards prostaglandins (PGs), which reinforce senescence [7]. As an alternative, free AA can be activated by esterification with coenzyme A (CoA) to form AA-CoA via ACSL4, also known as fatty acid-CoA ligase 4 (FACL4). ACSL4 is an AA-selective LC-acyl-CoA synthase, which can support mitochondrial integrity and, conversely, promote cell death by ferroptosis [9]. In addition to being a precursor in FA metabolism, the AA-CoA ester can participate in cellular Ca^2+^ signaling [10–12]. Senescent cells depend on excessive flux of Ca^2+^ from the endoplasmic reticulum (ER) to the mitochondria [13]. To prevent Ca^2+^ overload, which may cause cell death, senescent cells temporarily open the mitochondrial permeability transition pore (mPTP) [13]. This process, which is mediated by cyclophilin D [13], facilitates the release of mitochondrial DNA (mtDNA) into the cytosol, thereby triggering the cyclic GMP-AMP synthase and stimulator of interferon genes (cGAS-STING) pathway, which contributes to the senescence-associated secretory phenotype (SASP) [14]. In addition to its role as a DNA sensor, STING functions as a lipid sensor regulating the production of cholesterol and PUFAs in response to lipid accumulation [15] and as a proton channel, initiating autophagy and maintaining organelle homeostasis [16]. In the TME, activation of the cGAS-STING pathway restricts the mobility of TAMs [17]. TAMs can display strong features of senescence [18], although the identification of bona fide senescent macrophages is difficult [19] due to the close interplay between monocytes or macrophages and senescent cells, coupled with the overlap of characteristics [20]. As a result, macrophage senescence is less well studied compared to other cell types and a framework for understanding the development of pathogenic human macrophages, including TAMs, is missing.

The orphan nuclear receptor NR4A1 (Nur77, TR3, NGFI-B) governs macrophage M2 polarization [21]. In pathogenic IL-1ß^+^ macrophages, NR4A1 is one of several transcription factors driving the expression of a distinct gene signature synergistically induced by tumor necrosis factor α (TNF) and PGE_2_ [2, 22]. NR4A1 shares mechanistic similarities with p53 including the ability to regulate mitochondrial dynamics and determine cell fate, such as death and survival [23, 24]. The role of NR4A1 extends from causing mitochondrial disruption [25] to restoring homeostasis through the promotion of mitophagy [26]. NR4A1 plays a key role in the response to AA. Binding of AA to the ligand-binding domain of NR4A1 supports its stabilization [27]. In addition, the expression of *NR4A1* may directly result from ACSL4 activity [28].

In this study, we observed that primary human macrophages undergoing M2 polarization keep the expression of p53 and NR4A1 low through autocrine macrophage migration inhibitory factor (MIF) signaling. Using a three-pronged approach integrating transcriptional, secretory, and cell surface phenotyping, we show that de-repression of p53 and NR4A1 following disruption of MIF signaling drives macrophages toward a phenotype consistent with cellular senescence that resembles TAMs identified by scRNA-seq across most human cancers. [2–4]. In these TAM-like macrophages, ACSL4 ensured cell survival, prevented IL-1 cytokine release and instead fueled pathogenic inflammation. The CDK4/6 inhibitor abemaciclib (ABE) shifted the balance towards the inflammatory TAM phenotype by suppressing ACSL4 off-target, which explains the indirect anti-tumor effects of ABE recently observed in both murine tumor models and clinical cancer trials [29–31].

## Results

### Human macrophages on their path to the M2 phenotype utilize autocrine MIF signaling to keep p53 low

**Fig. 1:**
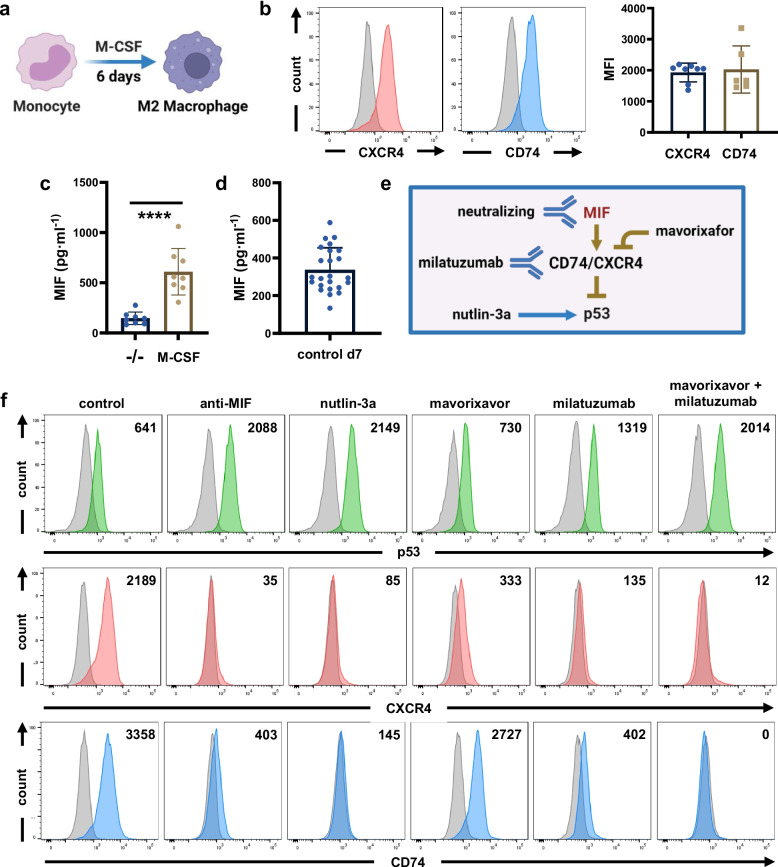
Autocrine MIF signaling keeps p53 low during M2 polarization of human monocyte-derived macrophages. **a** Schematic representation of the experimental cell system: M2-like human macrophages differentiating from monocytes in the presence of M-CSF. Created in BioRender. Thurnher, M. (2026) https://BioRender.com/bz6myr9**b** Flow cytometric detection of the MIF receptor components, CD74 and CXCR4 (representative histogram). Quantification of CD74 (*n* = 6) and CXCR4 (*n* = 8) expression (mean fluorescence intensities, MFI). **c** ELISA-determined spontaneous and M-CSF induced MIF release from monocytes (*n* = 8; unpaired *t* test). **d** MIF in culture medium of d6 macrophages replated overnight at 150.000 cells in 300 µL in the absence of M-CSF (*n* = 24). **e** Scheme illustrating the experimental approach to MIF neutralization and blockade of MIF receptor components. Created in BioRender. Thurnher, M. (2026) https://BioRender.com/99dupwv**f** Flow cytometric assessment of p53, CXCR4 and CD74 expression with or without antibody-mediated MIF depletion, nutlin-3a-mediated p53 activation or blockade of MIF receptor components (milatuzumab, mavorixafor). Day-6 M-CSF-differentiated macrophages treated with either vehicle or an isotype-matched irrelevant antibody served as the control. Representative flow cytometry histograms are shown. Statistical analyses (one-way ANOVA) were performed on data from *n* ≥ 3 independent experiments. Corresponding MFI values and *p*-values are provided in Supplementary Material 2

Building on our previous identification of C-X-C chemokine receptor 4 (CXCR4) as a regulator in human monocyte-derived macrophages [32], we found that macrophage colony-stimulating factor (M-CSF)-induced M2-like macrophages (Fig. 1a and Fig. S1a, Supplementary Material 1) expressed both CD74 and CXCR4 (Fig. 1b), which can form a heterodimeric receptor for MIF [33]. Consistent with active MIF signaling, monocytes secreted MIF spontaneously, a process that was enhanced by M-CSF (Fig. 1c) and maintained after re-plating of day-6 macrophages in M-CSF-free medium (Fig. 1d). MIF counteracts p53 [34], limiting its brake on M2 polarization [5]. Consistently, intracellular flow cytometry showed low p53 in day-6 M-CSF macrophages. However, MIF neutralization or mouse double minute 2 homolog (MDM2) inhibition using nutlin-3a (Fig. 1e) increased p53 (*p* < 0.007) (Fig. 1f). Milatuzumab (anti-CD74) or mavorixafor (CXCR4 antagonist) alone had relatively little effect, but together synergistically upregulated p53 (*p* = 0.014) to levels seen with MIF neutralization or nutlin-3a (Fig. 1f), confirming CD74/CXCR4 as the primary MIF receptor in human monocyte-derived macrophages [33]. MIF neutralization, nutlin-3a, or combined milatuzumab/mavorixafor abolished CD74 and CXCR4 expression (*p* < 0.005) (Fig. 1f), consistent with p53 accumulation removing the MIF receptor and overcoming MIF-mediated p53 suppression.

To evaluate macrophage reprogramming after MIF depletion or p53 activation, we performed NanoString-based gene expression analysis. We first validated the robustness of this technology by comparing current data from untreated M-CSF macrophages with a prior dataset generated three years earlier [35]. Comparison of the two datasets showed identical top five genes and a total of 12 shared genes among the top 20 (Fig. S1b-c). The presence of *CD68* and *CD163* among the top 20 overexpressed genes confirmed macrophage identity (Fig. S1a-c), while the absence of T-cell genes confirmed sample purity (Fig. S1d).

Extending our previous work [32], we found that MIF depletion induced macrophage secretion of CC-chemokine ligand 20 (CCL20) (MIP-3α/LARC) (Fig. S2), a p53-dependent SASP factor [36–39] linked to mitochondrial stress. Notably, TAM-derived CCL20 promotes tumor progression by acting on tumor cells and impairing immune surveillance [40–42]. CCL20 induction by MIF depletion was reduced by the p53 transcription inhibitor pifithrin (PFT)-α and strongly suppressed by PFT-µ (Fig. S2a), which blocks p53 mitochondrial binding [43]. Nutlin-3a also induced CCL20 production. Milatuzumab alone induced CCL20 secretion, while mavorixafor alone was ineffective but synergistically enhanced the milatuzumab response (Fig. S2b). MIF can also function intracellularly [44]. Consistent with a suppressive role of intracellular MIF, the MIF inhibitor 4-Iodo-6-phenylpyrimidine (4-IPP) enhanced CCL20 induction by MIF depletion or nutlin-3a, while the iron chelator deferioxamine (DFO), which causes mitochondrial dysfunction, further potentiated CCL20 production under both conditions (Fig. S2c-d). TC14012, a CXCR7 agonist [45], can costimulate CCL20 production in macrophages by suppressing CXCR4 [32]. In line, TC14012 boosted CCL20 production induced by MIF depletion or nutlin-3a treatment (Fig. S2e). Moreover, consistent with CXCR7’s ability to act as an antagonist of CXCR4, TC14012 suppressed macrophage MIF secretion (Fig. S2f). C–C Motif Chemokine Receptor 6 (*CCR6*), the sole receptor for CCL20, was not detected in control or p53-activated macrophages (*CCR6* mRNA copy number: 5; threshold: 60), ruling out autocrine or paracrine CCL20 signaling.

### MIF depletion promotes the development of known TAM phenotypes

**Fig. 2:**
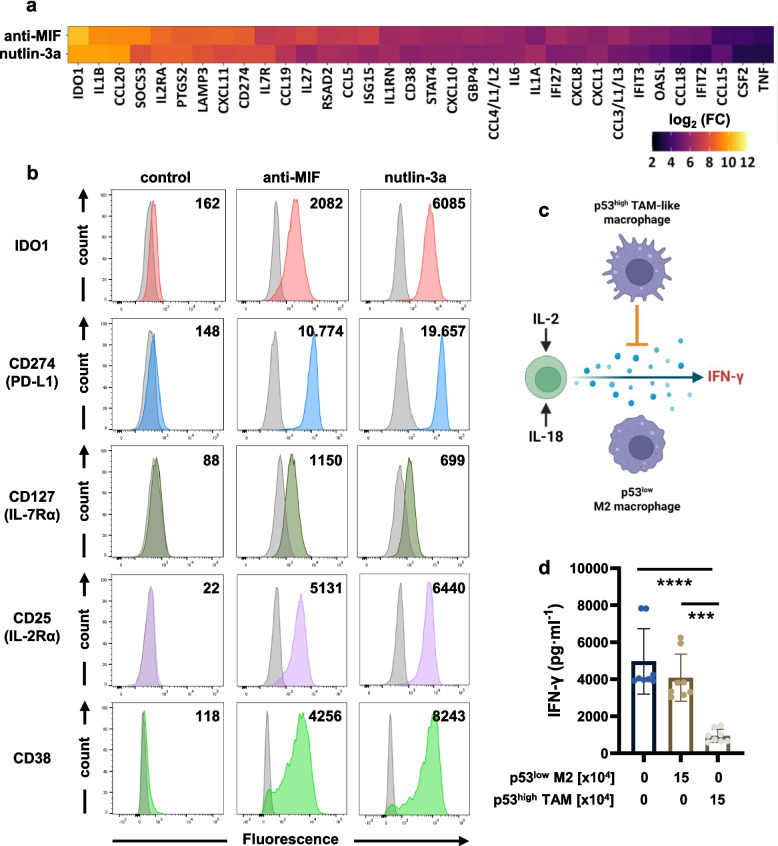
MIF depletion induces reprogramming of human macrophages and gives rise to a TAM-like phenotype. **a** Heatmap representing the top 20 genes significantly overexpressed (*n* = 3; *p* < 0.05) in either MIF-depleted or nutlin-3a treated, i.e. p53-activated, human macrophages as assessed by NanoString-based gene expression analysis using the Host Response Panel. **b** Flow cytometric analysis to control whether the transcriptional phenotype of anti-MIF or nutlin-3a treated macrophages translates into the corresponding surface phenotype. Representative flow cytometry histograms are shown. Statistical analyses (one-way ANOVA) were performed on data from *n* ≥ 3 independent experiments. Corresponding MFI values and *p*-values are provided in Supplementary Material 2. **c** Schematic representation of TAM-like macrophage-mediated immunosuppression. Created in BioRender. Thurnher, M. (2026) https://BioRender.com/z2ywlcu**d** CBA-based assessment of IFN-γ in co-cultures of macrophages (control versus nutlin-3a treated) and CD14-depleted PBMCs (containing T and NK cells) treated for 24 h with recombinant IL-2 (100 U/ml) and IL-18 (200 ng/ml) (*n* = 4; one-way ANOVA)

Using a three-pronged approach (Fig. S3a), with CCL20 as a surrogate marker of MIF depletion and p53 activation (Fig. S3b), NanoString analysis showed widespread gene upregulation and near-identical expression profiles between MIF depletion and nutlin-3a treatment (80% overlap among top 50 genes), while addition of PFT-µ to anti-MIF neutralizing antibody (anti-MIF) apparently caused large-scale changes in gene expression (Fig. S3c), emphasizing the importance of the p53 mitochondrial pathway. The genes in Fig. 2a were significantly upregulated (*p* < 0.05) and represent the top 20 by fold change (FC) in at least one treatment group, with *IDO1* (encoding indoleamine 2,3-dioxygenase 1), *IL1B*, and *CCL20* among the top three in both panels. MIF-depleted p53^high^ macrophages showed a transcriptional profile combining features of TAMs, especially resembling the IL-1β^+^ TAMs (*IL1B*, *TNF*, *PTGS2*) identified across multiple cancers, including renal cancer [2, 4].

In addition, the transcriptional phenotype of MIF-depleted p53^high^ macrophages showed a strong overlap with the phenotype of IL4I1^+^ TAMs, which exhibit the highest level of *CD38* expression of all subsets analyzed [3]. The IL4I1^+^ TAM marker *LAMP3* was also upregulated.

MIF-depleted p53^high^ TAM-like macrophages showed strong induction of interferon (IFN)-stimulated genes (*ISG15*, *IFI27*, *IFIT2*, *IFIT3*) and shared features with chronic inflammatory TPP macrophages [46], including expression of *IDO1*, *CD25*, and *PTGS2*. TPP macrophages develop in response to TNF, PGE_2_ and a TLR2 ligand, similar to IL-1ß^+^ TAMs [2]. Consistently, signal transducer and activator of transcription 4 (*STAT4*), a key regulator of TNF/PGE_2_-driven inflammation [46], was strongly upregulated (Fig. 2a). Gene expression was further aligned with previously defined monocyte/macrophage clusters for comparison (Fig. S4a–e).

Most top upregulated genes overlapped with senescence-associated signatures [37–39] (SeneQuest: available at https://senesquest.net) [39], consistent with transcriptional activation of the senescence-associated secretome [47], with approximately 50% encoding cytokines, chemokines, or growth factors. In contrast, only one cytokine (*IL16*) was downregulated (Fig. S5a). Both MIF depletion and nutlin-3a induced ETS proto-oncogene 1 (*ETS1*) upregulation and cyclin-dependent kinase 4 (*CDK4*) downregulation (Fig. S5b). ETS-1, a senescence-associated transcription factor, promotes p16INK4A (inhibitor of CDK4) expression and CCL20 induction, linking it to cell cycle arrest [48–50]. However, senescence markers such as p16INK4A and senescence-associated β-galactosidase (SA-ß-Gal) are also induced during physiological M2 macrophage activation, complicating senescence detection [51]. Accordingly, M-CSF-differentiated macrophages showed basal SA-β-Gal levels that decreased upon p53 activation (Fig. S5c), consistent with p53 acting as a brake on M2 polarization [5].

RayBio-based semi-quantitative secretome analysis (Fig. S6a) revealed a cytokine profile resembling that reported for IL-1β^+^ TAMs (CCL3, CXCL1, VEGF, TNF) [2] and IL4I1^+^ TAMs (CCL8, CXCL9, CXCL10, CXCL11) [3]. In addition to being part of TAM secretomes, the soluble factors produced by MIF-depleted p53^high^ macrophages (Fig. S6a) are typical SASP components [37–39], in line with the fact that TAMs often exhibit a senescent phenotype [18]. The secretion of C-X-C motif chemokine ligand 10 (CXCL10) and soluble TNF receptor II (TNFR II) was further validated (Fig. S2g). In contrast, *IL1B*, *CSF2* (encoding granulocyte–macrophage colony-stimulating factor; GM-CSF), CC-chemokine ligand 4 (*CCL4*) and *IL27* could not be validated at the protein level, and IL-1β secretion was unexpectedly absent despite a strong IL-1/IL-1 receptor transcriptional signature in MIF-depleted p53^high^ macrophages (Fig. S5d).

Flow cytometry was used to validate the TAM-like transcriptional phenotype at the protein level (Fig. 2b) and to confirm that CD40, another marker of IL4I1^+^ TAMs (Fig. S4d) [3], was upregulated along with CD38 (Fig. S6b).

IDO1 inhibition with epacadostat reduced its expression (Fig. S6c), suggesting a self-sustaining IDO1 loop that reinforces the immunosuppressive TAM-like phenotype. Although CD25 (IL-2Rα) could imply IL-2 scavenging, there was no evidence of uptake of recombinant IL-2 or of its effect on the secretome of TAM-like macrophages (Fig. S6d–e).

To assess the immunoregulatory function of p53^high^ TAM-like macrophages, we tested their impact on lymphocyte activation by measuring IL-2/IL-18-induced IFN-γ production [52] in CD14-depleted peripheral blood mononuclear cells (PBMCs) (Fig. 2c). This setup confirmed the strong suppressive capacity of TAM-like macrophages (Fig. 2d).

**Fig. 3:**
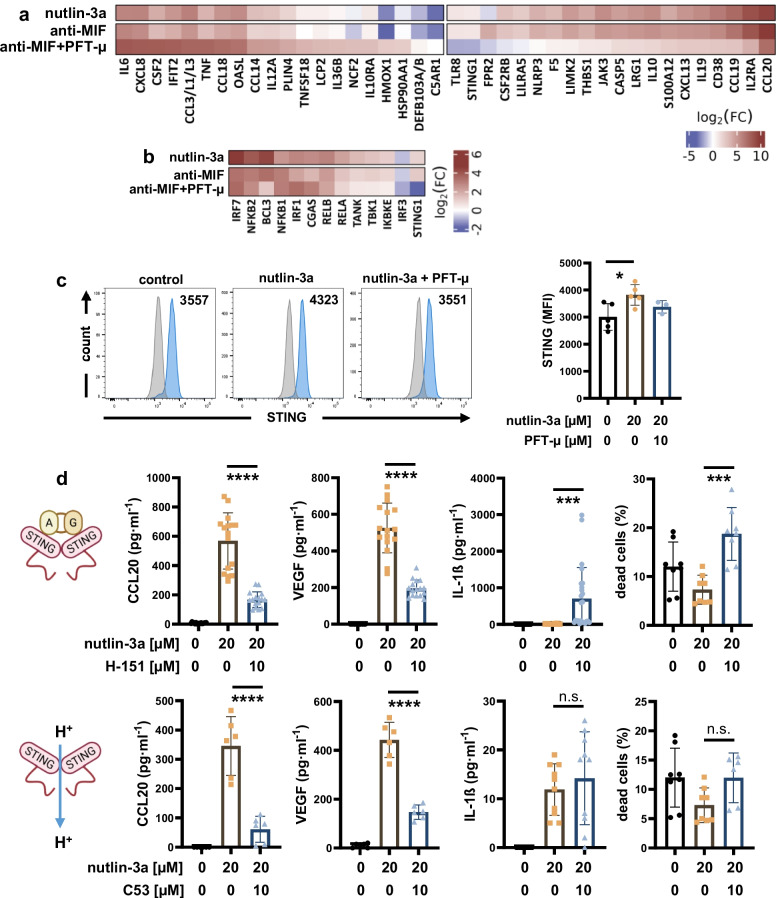
Mitochondrial p53 promotes an anti-inflammatory program involving STING in human TAM-like macrophages. **a** Heatmap showing the 20 genes that were most overexpressed (left panel) and the 20 genes that were most underexpressed (right panel) as a result of treatment with anti-MIF plus PFT-µ as compared to anti-MIF alone (*n* = 3; *p* < 0.05). The genes were plotted as FC over control. **b** Heatmap showing upregulation of genes constituting the cGAS-STING pathway as well as gene regulation by PFT-µ (*n* = 3; *p* < 0.05). **c** Flow cytometric validation of STING protein expression and effects of PFT-µ. Representative flow cytometry histograms are shown. Statistical analyses (one-way ANOVA) were performed on data from *n* ≥ 3 independent experiments. Corresponding MFI values and *p*-values are provided in Supplementary Material 2. **d** Measurement of SASP components using ELISA and cytokine bead arrays (CBA) in the cell culture medium of nutlin-3a treated macrophages versus control macrophages (*n* ≥ 3; one-way ANOVA). Dead cells were quantified by flow cytometry using fixable viability dye eFluor 780 (*n* = 3). The role of STING was examined using the inhibitors, H-151 (covalent STING antagonist) and C53 (inhibitor of STING’s proton channel activity). Schematic representation of STING: Created in BioRender. Thurnher, M. (2026) https://BioRender.com/huss17j

Because CCL20 production depended strongly on mitochondrial p53 (Fig. S2a) [36], we examined whether PFT-µ alters transcriptional reprogramming in MIF-depleted TAM-like macrophages. Differential gene expression analysis identified the 20 most up- and downregulated genes (*p* < 0.05) in anti-MIF + PFT-µ versus anti-MIF alone, plotted as FC over control (Fig. 3a). This separated the gene profile of TAM-like macrophages into a pro-inflammatory signature (left panel: *IL6*, *CXCL8*, *TNF*) and an anti-inflammatory/repair-associated program (right panel: *IL10*, *IL19*, *CD38*, *CCL20*).

### The cGAS-STING pathway is enhanced in human TAM-like macrophages

The anti-inflammatory gene set also contained *STING* (Fig. 3a, right panel), a p53 target [53, 54] linked to senescence and aging [14, 55]. Consistently, other components of the cGAS-STING pathway were activated upon MIF depletion or nutlin-3a treatment (Fig. 3b). Strongly upregulated genes (Fig. 2a) further included STING downstream effectors such as immune checkpoints (*CD274*, *IDO1*) [14, 56], cytokines and chemokines (*IL1B*, *IL6*, *CXCL8*, *CXCL10*, *CCL20*) [53, 55, 57], IFN-stimulated genes (*ISG15*, *RSAD2*, *IFI27*, *IFIT2*, *IFIT3*) [14] along with cGAS-STING pathway regulators (*SOCS3*, *OASL*) [58, 59]. In parallel, key RNA sensors (*DDX58*/*RIG-I*, *IFIH1*/*MDA5*, *DHX58*, *EIF2AK2*/*PKR*) and the dsRNA-modulating enzyme *ADAR* were also increased (Fig. S7a). Notably, *RIG-I* and *MDA5* have been implicated in senescence-associated inflammation via sensing mitochondrial dsRNA [60].

Intracellular flow cytometry confirmed STING expression in human M-CSF macrophages (Fig. 3c and Fig. S7B). Although *STING* mRNA increased by more than twofold (*p* < 0.007) following MIF depletion or nutlin-3a treatment, protein levels increased only modestly (*p* = 0.0251) and blockade of STING upregulation by PFT-µ was not significant. To assess STING’s role in the secretory phenotype of TAM-like macrophages, we used two inhibitors: H-151, a covalent STING antagonist [61], and C53, which blocks STING’s proton channel activity [62]. H-151 markedly reduced CCL20 and vascular endothelial growth factor (VEGF) production (Fig. 3d) but had little effects on other SASP factors (Fig. S7c). Interestingly, H-151 increased IL-1β production—albeit with high donor variability (Fig. 3d), suggesting STING helps restrain IL-1β, consistent with an anti-inflammatory, homeostatic function (Fig. 3a) [16]. Like H-151, C53 selectively reduced nutlin-3a-induced CCL20 and VEGF, but did not elevate IL-1β (Fig. 3d). STING inhibition also induced cell death, reversing the protective effect of p53, again supporting a cytoprotective role of STING. The strong activation of DNA and RNA sensing pathways in TAM-like macrophages points to mitochondrial permeability transition, in line with p53-mediated pore opening [63] and release of mtDNA and mtRNA.

Transient mPTP opening via cyclophilin D acts as a failsafe to prevent cell death under Ca^2^⁺ overload, particularly in senescent cells [13, 64]. To test this, we inhibited cyclophilin D with cyclosporin A (CsA). CsA induced significant cell death selectively in p53^high^ TAM-like macrophages, but not in p53^low^ controls (Fig. S8a). Dying TAM-like macrophages released IL-1α and IL-1β (Fig. S8b), suggesting an inflammatory form of cell death. However, only cytokine release was suppressed by STING inhibitors H-151 and C53 (Fig. S8b). STING inhibition did not prevent CsA-induced cell death (Fig. S8a, left panel). Because CsA also targets calcineurin, we used NIM811, a derivative lacking calcineurin inhibition [65]. NIM811 induced cell death as effectively as CsA (Fig. S8a, right panel), indicating that cyclophilin D-mediated mPTP opening is essential for the survival of p53^high^ TAM-like macrophages.

### TAM-like macrophages activate ACSL4 to avoid cell death and prevent STING-dependent IL-1 cytokine release

To cope with high levels of unesterified AA, for instance during senescence, cells can upregulate *PTGS2* (encoding COX-2). Consistently, TAM-like macrophages showed strong induction of *PTGS2* (Fig. 2a), which is a p53 target in senescence [7] and a hallmark of IL-1β^+^ TAMs and TPP macrophages (Fig. S4c, e). The gene profile of TAM-like macrophages indicated a clear shift of AA metabolism toward PG synthesis (Fig. S9a-b). Notably, *STAT4* was also activated (Fig. S9a), aligning with a chronic inflammatory TP/TPP phenotype [22, 46]. The selective COX-2 inhibitor celecoxib significantly reshaped the secretome of p53^high^ TAM-like macrophages, mainly by reducing VEGF and CCL20 secretion (Fig. S9c). While concentrations up to 40 µM did not trigger IL-1 cytokine release (Fig. S9c, Supplementary Material 3), celecoxib did cause some cell death (Fig. S9d).

**Fig. 4:**
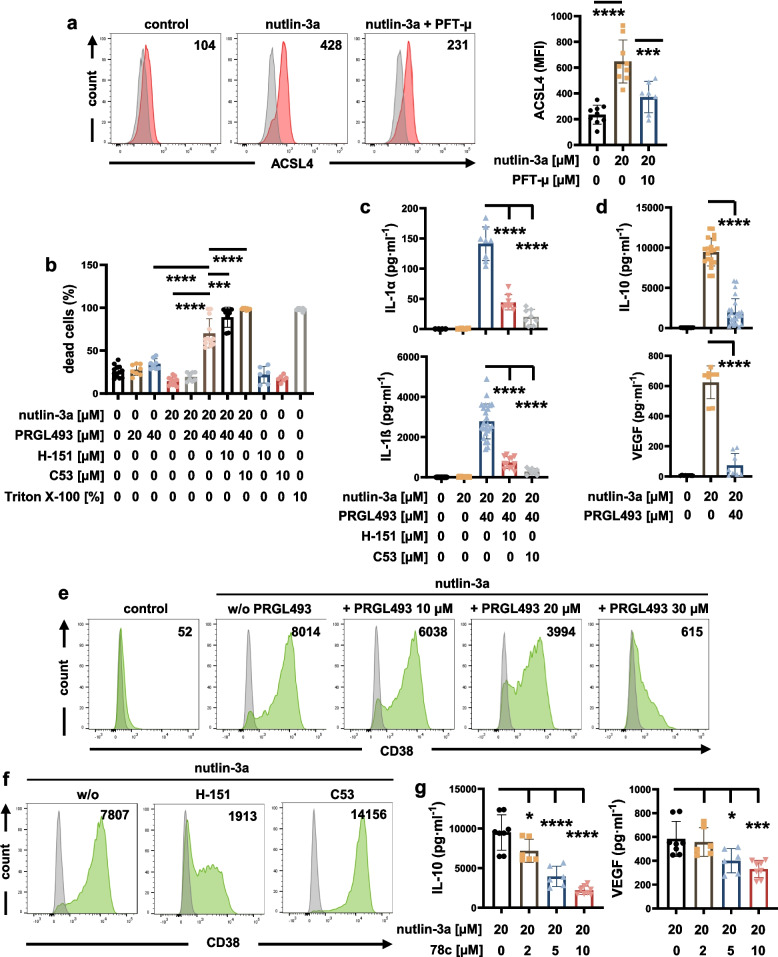
p53 engages ACSL4 to prevent cell death and STING-mediated inflammation in human TAM-like macrophages. **a** Intracellular flow cytometric validation of ACSL4 protein expression in TAM-like macrophages versus control macrophages and its regulation by PFT-µ. Representative histograms and quantification (*n* ≥ 7; one-way ANOVA). **b** Effect of ACSL4 inhibition using PRGL493 on the survival of nutlin-3a treated versus control macrophages. Dead cells were quantified by flow cytometry using fixable viability dye eFluor 780. The role of STING was examined using the inhibitors, H-151 (covalent STING antagonist) and C53 (inhibitor of STING’s proton channel activity) (*n* ≥ 4; one-way ANOVA). The membrane disrupting non-ionic surfactant Triton X-100 was used to induce maximal cell death. **c** CBA-based measurement of IL-1α and IL-1ß induced by ACSL4 inhibitor PRGL493 in nutlin-3a-activated versus control macrophages. H-151 and C53 were used to investigate the role of STING (*n* ≥ 4; one-way ANOVA). **d** CBA-based measurement of IL-10 (*n* = 6) and VEGF (*n* = 4) released from nutlin-3a-activated versus control macrophages and its regulation by the ACSL4 inhibitor PRGL493 (one-way ANOVA). **e** Flow cytometric analysis of CD38 protein expression on nutlin-3a-activated versus control macrophages and dose-dependent regulation by ACSL4 inhibitor PRGL493. **f** Regulation of CD38 expression by the STING inhibitors, H-151 and C53, in TAM-like macrophages. **e–f** Representative flow cytometry histograms are shown. Statistical analyses (one-way ANOVA) were performed on data from *n* ≥ 3 independent experiments. Corresponding MFI values and *p*-values are provided in Supplementary Material 2. **g** CBA-based measurement of IL-10 and VEGF released from nutlin-3a treated macrophages and regulated by the CD38 inhibitor 78c (*n* = 4; one-way ANOVA)

*ACSL4*, encoding another metabolic enzyme with marked preference for AA [7, 66], was also upregulated in TAM-like macrophages in a manner dependent on mitochondrial p53 (anti-MIF: 8.9-fold, *p* = 0.0027; anti-MIF + PFT-µ: 1.79-fold, *p* = 0.2242; nutlin-3a: 4.91-fold *p* = 0.0048). We confirmed ACSL4 protein upregulation and its dependence on mitochondrial p53 by flow cytometry (Fig. 4a; Fig. S10a). For functional studies, we used the ACSL4 inhibitor PRGL493 [67], which mimics the effects of gene silencing, as ACSL4 knockdown and inhibiting ACSL4 activity with PRGL493 were shown to downregulate a shared set of genes, including NR4A1 [28]. Strikingly, PRGL493 induced extensive cell death specifically in p53^high^ TAM-like macrophages, while p53^low^ control cells were largely unaffected (Fig. 4b; Fig. S10b). In TAM-like macrophages, ACSL4 inhibition not only triggered cell death but also IL-1α and IL-1β release (Fig. 4c). IL-1β, but not IL-1α, depended on caspase-1 (Fig. S10c), while both cytokines required STING receptor and proton channel activity (Fig. 4c). As seen with CsA (Fig. S8a), STING inhibition did not prevent but instead increased ACSL4 inhibitor-induced cell death (Fig. 4b). Although *ACSL1*, which contributes to the synthesis of AA-CoA [68] was also upregulated (Fig. S10d), its inhibition with triacsin C (selective for ACSL1 over ACSL4) [69] led to only minimal IL-1 cytokine release (Fig. S10e). These findings collectively indicate that ACSL4 is essential for the survival of TAM-like macrophages and that ACSL4 preserves a homeostatic STING function that prevents IL-1 cytokine release.

### ACSL4 drives the expression of CD38 in TAM-like macrophages

Given the strong impact of ACSL4 inhibition on viability (Fig. 4b), we examined how PRGL493 affects the secretory and surface phenotype of TAM-like macrophages. PRGL493 markedly reduced IL-10 and VEGF secretion (Fig. 4d). Recombinant IL-2 (via CD25) and IL-7 (via CD127/IL-7Rα) showed minimal protective effects (Fig. S11a). The caspase-1/4 inhibitor VX-765 [13] also failed to prevent ACSL4 inhibition-induced cell death, and ferroptosis or necroptosis inhibitors provided only limited rescue (Fig. S11a).

Regarding the TAM-like surface phenotype, PRGL493 dose-dependently blocked CD38 upregulation (Fig. 4e; Fig. S11b), a marker of immunosuppressive IL4I1⁺ TAMs [3]. STING has also been linked to CD38 expression [70]. Consistently, CD38 induction was also prevented by H-151, while the STING proton channel inhibitor C53 instead increased CD38 expression (Fig. 4f), although this was not significant due to donor variability.

To investigate the purpose of CD38 upregulation, we used 78c, a selective inhibitor of CD38-mediated NAD⁺ degradation. The main effect of 78c on the TAM-like macrophage secretome was reduced IL-10 production and, to a lesser extent, VEGF (Fig. 4g). Recombinant IL-10 did not restore cell survival (Fig. S11c) but reduced IL-1α and IL-1β release (Fig. S11d). The fact that the receptor function of STING prevents IL-1ß secretion (Fig. 3d) and promotes CD38 expression (Fig. 4f), suggested that the CD38-IL-10 axis is responsible for IL-1ß suppression. In line, neutralization of IL-10 in combination with 78c-mediated CD38 inhibition was sufficient to induce IL-1ß secretion in TAM-like macrophages (Fig. S11e). IL-6 and CCL20 also increased in the presence of 78c, while TNF was largely unchanged (Fig. S12a).

ACSL4 generates AA-CoA, which can enter FA oxidation or contribute to lipid synthesis. FA synthase (FASN) is known to increase during senescence and to be required for its induction [8]. Consistently, inhibition of FASN with C75 (10 µM) strongly reduced the nutlin-3a-induced secretome (Fig. S12b), validating the senescent phenotype of TAM-like macrophages. In contrast, blocking FA oxidation with etomoxir had little or no effect.

### Abemaciclib targets the ACSL4-CD38 pathway in human TAM-like macrophages

**Fig. 5:**
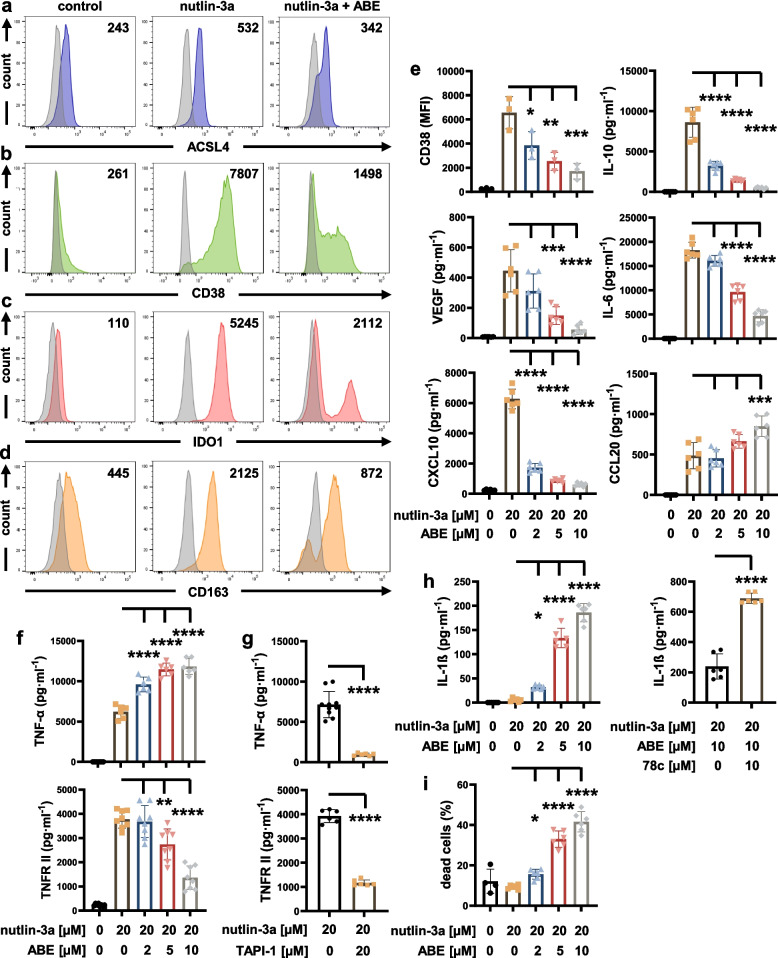
ABE suppresses the ACSL4-CD38 pathway in human TAM-like macrophages and causes macrophage repolarization. **a-d** Flow cytometric analysis of ACSL4 (intracellular), CD38 (surface), IDO1 (intracellular) and CD163 (surface) in TAM-like macrophages and regulation by the CDK4/6 inhibitor ABE (10 µM). Representative flow cytometry histograms are shown. Statistical analyses (one-way ANOVA) were performed on data from *n* ≥ 3 independent experiments. Corresponding MFI values and *p*-values are provided in Supplementary Material 2. **e** Dose response analysis of ABE: flow cytometric (CD38), CBA-based (IL-10, VEGF, IL-6, CXCL10) and ELISA-based (CCL20) assessment in nutlin-3a-activated versus control macrophages with or without treatment with ABE (*n* = 3; one-way ANOVA). **f** CBA-based assessment of TNF and soluble TNFR II in nutlin-3a-activated macrophages with or without treatment with ABE (2, 5, 10 µM) (*n* = 3; one-way ANOVA). **g** Effects of ADAM17 inhibitor TAPI-1 (20 µM) on TNF (*n* = 3)/TNFR II (*n* = 4) ectodomain shedding (unpaired *t* test). **h** Left panel: CBA-based measurement of IL-1ß in nutlin-3a-activated versus control macrophages with or without treatment with ABE (*n* = 3; one-way ANOVA). Right panel: in the presence or absence of CD38 inhibitor 78c (*n* = 3; unpaired *t* test). **i** Dead cells were quantified by flow cytometry using fixable viability dye eFluor 780 (*n* = 3; one-way ANOVA)

The CDK4/6 inhibitor abemaciclib (ABE) can induce a p53-dependent senescent state [71], underlying its therapeutic efficacy in cancer. Beyond its direct effects on tumor cells, ABE has also been shown to stimulate anti-tumor immunity [29], although the mechanisms remain unclear. In a machine-learning-based drug discovery approach, ABE was identified as a selective inhibitor of ACSL4 expression [72]. Consistent with this, ABE reduced nutlin-3a-induced ACSL4 upregulation in macrophages (Fig. 5a). In line with ACSL4 promoting CD38 expression, ABE also blocked CD38 induction (Fig. 5b). Unexpectedly, ABE additionally suppressed nutlin-3a-driven IDO1 and CD163 expression (Fig. 5c-d), suggesting macrophage reprogramming toward an altered polarization state.

In addition to the dose-dependent suppression of CD38 (Fig. 5e), ABE also reshaped the secretory profile of TAM-like macrophages. IL-10 and VEGF were reduced (Fig. 5e), consistent with inhibition of the ACSL4-CD38 axis (Fig. 5a-b). CXCL10, which is known to be involved in oncogenic signaling [73], was inhibited to the greatest extent. IL-6 also decreased, whereas CCL20 increased (Fig. 5e). One of the most striking effects of ABE was the dose-dependent increase in TNF secretion, accompanied by a decline in soluble TNFR II (Fig. 5f). TNFR II binds and neutralizes TNF, thereby limiting excessive inflammation. This observation was intriguing, because both, TNF and TNFR II release, depend on ADAM17 (a disintegrin and metalloprotease 17)-mediated ectodomain shedding. Consistently, ADAM17 inhibition with TAPI-1 reduced release of both TNF and TNFR II (Fig. 5g). ABE appears to differentially modulate ADAM17-dependent processing, thereby shifting the balance toward a pro-inflammatory state. ABE also enhanced IL-1β release, which was further increased by CD38 inhibition (Fig. 5h). In line with its suppression of ACSL4 (Fig. 5a), ABE induced cell death in TAM-like macrophages (Fig. 5i).

In the same machine-learning drug discovery approach that identified ABE, rosiglitazone (RSG) was also predicted to bind ACSL4 [72]. Despite having a lower docking score than ABE, RSG similarly suppressed nutlin-3a-induced CD38 upregulation and IL-10 expression (Fig. S13a-c), further supporting an ACSL4-CD38-IL-10 regulatory axis.

**Fig. 6:**
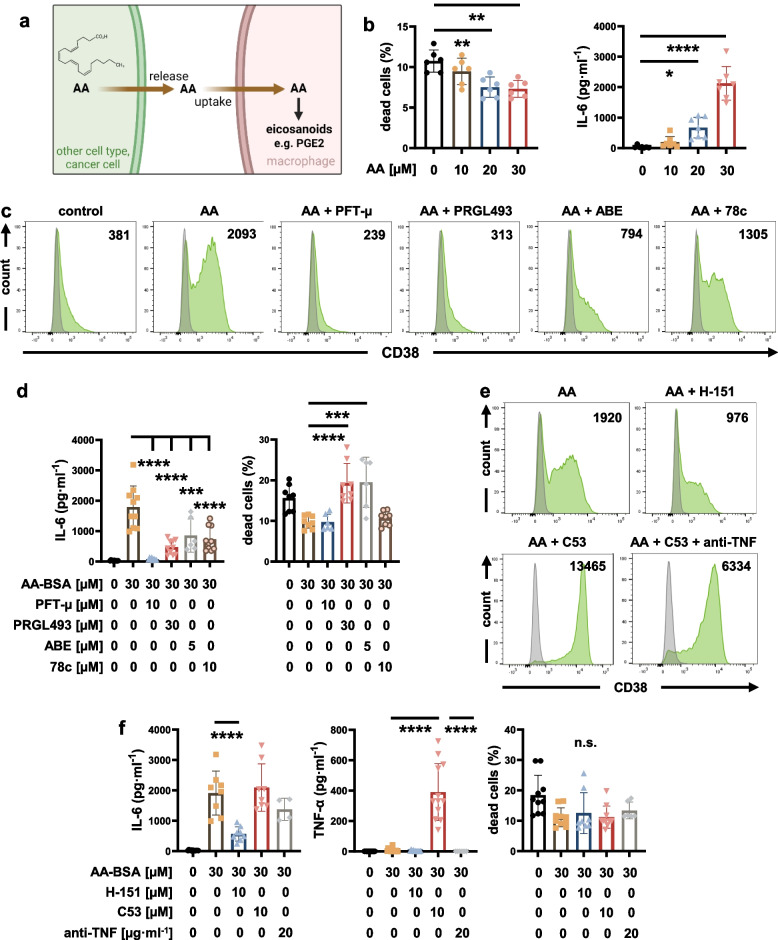
Macrophages activate the p53-ACSL4-CD38 axis and additionally engage the lipid sensor STING in response to AA exposure. **a** Schematic representation of transcellular AA metabolism. Created in BioRender. Thurnher, M. (2026) https://BioRender.com/i7jimyx**b** Treatment of control macrophages with increasing doses of AA-BSA and assessment of cell death using eFluor 780 and flow cytometry (*n* = 3) as well as CBA-based measurement of IL-6 (*n* = 5) (one-way ANOVA). **c** Flow cytometric analysis of CD38 expression induced by AA-BSA (30 µM) in control macrophages and its regulation by various inhibitors (*n* = 3; one-way ANOVA). Representative flow cytometry histograms are shown. Statistical analyses (one-way ANOVA) were performed on data from *n* = 3 independent experiments. Corresponding MFI values and *p*-values are provided in Supplementary Material 2. **d** CBA-based measurement of IL-6 (*n* ≥ 6) and flow cytometric assessment of dead cells using fixable viability dye eFluor 780 (*n* ≥ 3): regulation by inhibitors (one-way ANOVA). **e** Flow cytometric analysis of CD38 expression induced by AA-BSA (30 µM) in control macrophages and its regulation by STING inhibitors with our without TNF neutralizing antibody. Representative flow cytometry histograms are shown. Statistical analyses (unpaired *t* test) were performed on data from *n* ≥ 3 independent experiments. Corresponding MFI values and *p*-values are provided in Supplementary Material 2. **f** CBA-based assessment of IL-6 and TNF (*n* ≥ 4; one-way ANOVA)

Next, we performed AA loading experiments to examine how macrophages respond to exogenous AA. This sort of experiment also served to confirm that ACSL4 functions by forming AA-CoA rather than via unknown mechanisms. In addition, exogenous supply models transcellular AA metabolism (Fig. 6a) [74]. Because non-esterified FAs are physiologically transported by serum albumin, we used AA-BSA to enhance uptake by macrophages. Consistent with ACSL4’s role in macrophage survival (Fig. 4b), exogenous AA reduced spontaneous cell death in a dose-dependent manner (Fig. 6b). AA also induced the secretion of IL-6 (Fig. 6b) and CXCL8 (see below), while other SASP factors remained low or undetectable (Fig. 6b, Supplementary Material 2). AA also upregulated CD38 (Fig. 6c). These effects were blocked by PRGL493 and ABE (Fig. 6c-d), indicating that ACSL4-mediated AA conversion to AA-CoA is required. Inhibiting CD38 with 78c reduced both CD38 upregulation and IL-6 secretion (Fig. 6c-d), suggesting a self-sustaining loop. Notably, PFT-µ strongly suppressed AA-induced IL-6 production and CD38 expression, highlighting the role of mitochondrial p53 in this pathway. PRGL493 and ABE abolished the protective effect of AA on macrophages (Fig. 6d), which once again confirmed the importance of ACSL4 for macrophage survival.

In macrophages, STING acts as a lipid sensor that responds to FA overload by inducing IL-6 and TNF [75, 76]. Accordingly, AA-induced CD38 expression (Fig. 6e) and IL-6 production (Fig. 6f) depended on STING’s receptor activity (blocked by H-151). In contrast, inhibiting STING’s proton channel (C53) triggered TNF secretion (Fig. 6f) and significantly increased CD38 expression, an effect partially reduced by TNF neutralization (Fig. 6e). Inhibition of STING functions had no effect on macrophage viability (Fig. 6f).

### NR4A1 participates in TAM-like macrophage development

TAM-like macrophages exhibited elevated levels of TNF and PTGS2 (COX-2) (Fig. 2a), resembling TPP macrophages (Fig. S4e) [46], as well as IL-1β⁺ macrophages, which have been observed in pancreatic cancer and rheumatoid arthritis [2, 22]. These populations share a gene expression program driven by combined TNF and PGE₂ signaling. Consistent with this, the gene profile of TAM-like macrophages in our study showed significant overlap with the TP signature, encompassing *IL1A*, *IL1B*, *CD274*, *CXCL1*, *STAT4*, *PTGS2* and *IL2RA* (Fig. 2a).

**Fig. 7:**
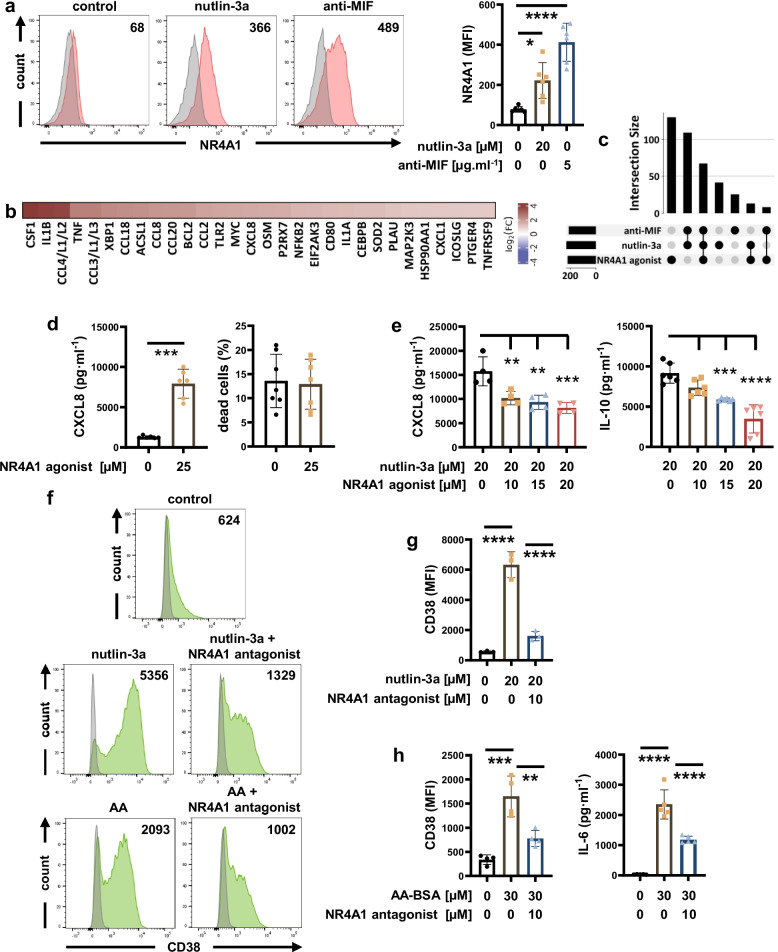
Nuclear receptor NR4A1 shapes TAM-like phenotype and function. **a** Flow cytometric assessment of NR4A1 expression with or without nutlin-3a treatment or antibody-mediated MIF depletion. Day-6 M-CSF-differentiated macrophages treated with either vehicle or an isotype-matched irrelevant antibody served as the control. Representative histograms and quantification (*n* = 6; one-way ANOVA). **b** Heatmap representing the top 30 genes significantly overexpressed in macrophages treated by NR4A1 agonist DIM-C-pPhOCH_3_ (25 µM). Gene expression was analyzed using NanoString technology (Host Response Panel) (*n* = 3; *p* < 0.05). **c** UPSET plot of gene intersections illustrating the overlap between the gene profiles activated by NR4A1 agonist, anti-MIF or nutlin-3a treatment. **d** CBA-based measurement of CXCL8 and flow cytometric assessment of dead cells using fixable viability dye eFluor 780 (*n* ≥ 3; one-way ANOVA). **e** CBA-based measurement of SASP components induced by nutlin-3a and regulated by NR4A1 antagonist DIM-C-pPhOH in a dose-dependent manner (*n* = 3; one-way ANOVA). **f** Flow cytometric analysis of CD38 protein expression on nutlin-3a-activated or AA-loaded macrophages and regulation by NR4A1 antagonist DIM-C-pPhOH. **g** Quantification of nutlin-3a induced and DIM-C-pPhOH-regulated CD38 expression (MFI) (*n* = 3; one way ANOVA). **h** Quantification of AA-BSA induced and DIM-C-pPhOH-regulated CD38 expression (*n* = 4; one way ANOVA). CBA-based measurement of IL-6 (*n* = 5; one-way ANOVA)

NR4A1 is a key regulator of M2 polarization [21] and one of several drivers of TP gene expression [22]. To assess its role in TAM-like macrophage development, we first confirmed NR4A1 protein expression and showed that it is also upregulated by nutlin-3a treatment and MIF depletion using intracellular flow cytometry (Fig. 7a). To examine its function, we analyzed gene expression in M2 macrophages treated with the NR4A1 agonist DIM-C-pPhOCH_3_ [77]. The top 30 upregulated genes are shown in Fig. 7b. The upset plot in Fig. 7c shows substantial overlap between genes induced by the NR4A1 agonist and those triggered by anti-MIF and nutlin-3a treatment. A total of 67 genes were overexpressed in all three treatment groups (Fig. 7c, Supplementary Material 2). The shared genes included not only the drivers of the TP signature itself (*TNF*, *PTGS2*, which encodes COX-2, and *PTGER4*, which encodes the PGE_2_ receptor EP4) [22], but also TP signature genes. The most notable of these was *IL1B*, the marker gene that defines IL-1β^+^ TAMs (Fig. 7b-c, Supplementary Material 2). *ACSL4* was also among the genes that were overexpressed in all three treatment groups.

*CSF1* (encoding M-CSF) was the most strongly upregulated gene following NR4A1 agonist treatment (Fig. 7b), consistent with its role in promoting the M2 phenotype [21]. *CXCL8*, a known M-CSF target [78], was also increased, and its protein secretion was confirmed (Fig. 7d). TNFR II, another SASP component constitutively produced by M-CSF macrophages (Fig. S6a and Fig. S2g), was likewise enhanced (Fig. S14a), while macrophage viability remained unaffected (Fig. 7d). *IL1B* was the second most upregulated gene, supporting NR4A1 as a key driver of the TP signature in IL-1β⁺ TAMs [22]. Consistently, *CEBPB*, which may cooperate with NR4A1, was also among the most overexpressed genes (Fig. 7b and Fig. S14a). In addition to *IL1B* and *CXCL8*, NR4A1 activation elevated the mRNA levels of other SASP components, including *TNF*, *CCL20*, *IL1A*, and *IL6* (Fig. 7b).

To confirm NR4A1’s functional role, we used the antagonist DIM-C-pPhOH (2–20 µM) [79], which blocks NR4A1 transactivation [77]. Consistent with NR4A1 agonist-driven CXCL8 gene and protein expression (Fig. 7b,d), DIM-C-pPhOH suppressed nutlin-3a-induced CXCL8 secretion (Fig. 7e). CCL20, VEGF and IL-6 secretion were also reduced, while TNF was only modestly affected (Fig. S14b). The most significant effect was the dose-dependent inhibition of IL-10 production (Fig. 7e), suggesting impaired CD38 upregulation. Indeed, DIM-C-pPhOH reduced nutlin-3a-induced CD38 expression, with first effects observed already at 2 µM (Fig. S14c) and near-complete inhibition at 10 µM (Fig. 7f-g). A slightly negative effect on cell viability was only observed at the highest concentration of DIM-C-pPhOH (20 µM) (Fig. S14b). NR4A1 is involved in the response to AA [27, 28]. Consistently, AA-BSA-induced CD38 upregulation and IL-6 production were significantly reduced by the NR4A1 antagonist DIM-C-pPhOH (Fig. 7h).

## Discussion

**Fig. 8:**
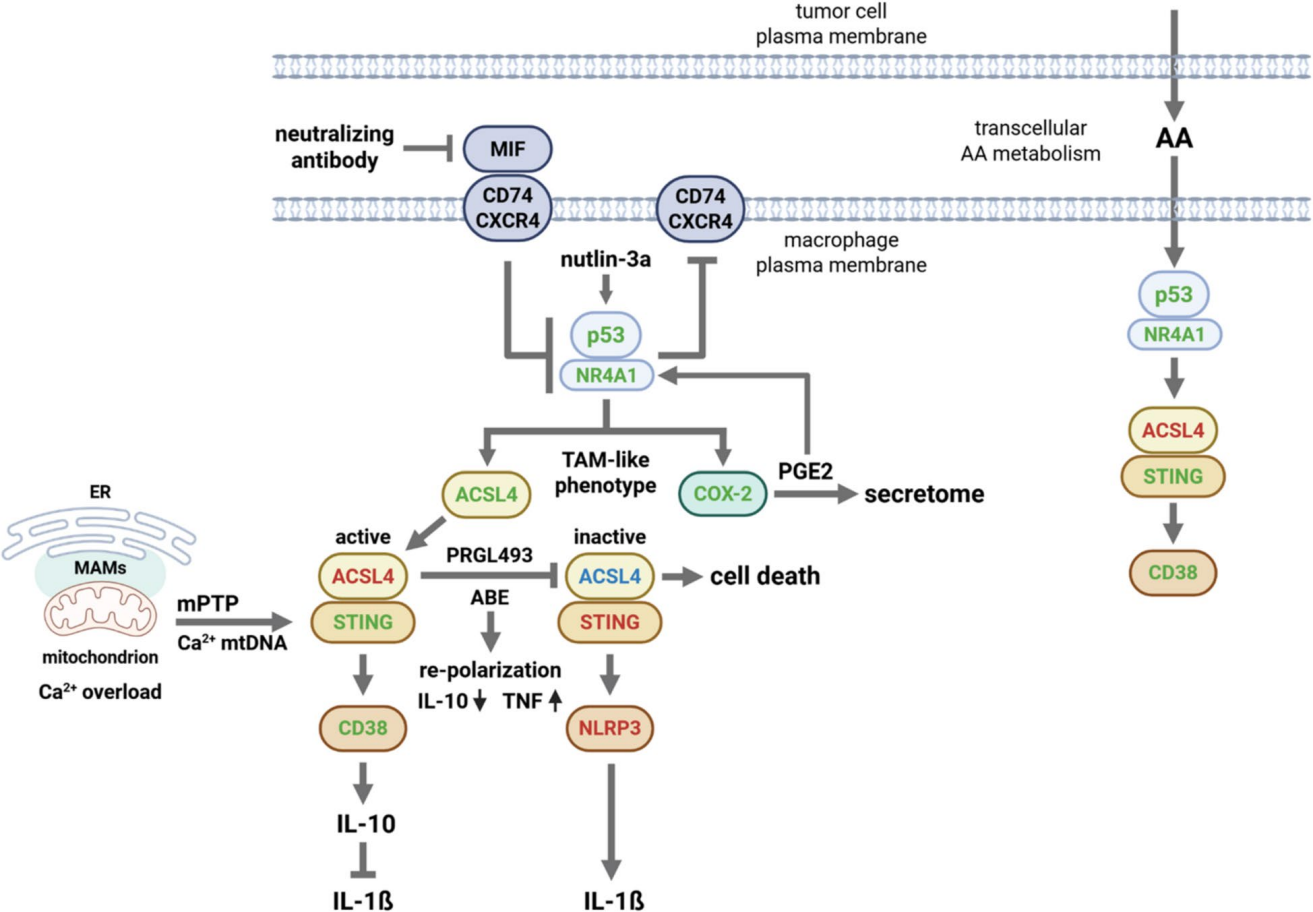
Framework for understanding the development of immunosuppressive human TAM-like macrophages from monocytes. Created in BioRender. Thurnher, M. (2026) https://BioRender.com/kweqe1m MIF signaling through its heterodimeric receptor (CD74/CXCR4) suppresses p53 during M2 macrophage differentiation. MIF depletion or nutlin-3a treatment de-represses p53 and co-activates NR4A1. The subsequent downregulation of the MIF receptor facilitates in a feed-forward manner the p53/NR4A1-driven development of a TAM-like phenotype. The AA-metabolizing enzymes, ACSL4- and COX-2, promote an anti-inflammatory TAM-like secretome. The clinical CDK4/6 inhibitor abemaciclib (ABE) can re-polarize TAM-like macrophages through off-target inhibition of an ACSL4-CD38-IL-10 axis. TAM-like senescent macrophages use cyclophilin D to open the mitochondrial permeability transition pore (mPTP). This releases excess Ca^2^⁺ and also mitochondrial DNA (mtDNA), which activates the cGAS-STING pathway. ACSL4 maintains the homeostatic, anti-inflammatory role of STING. Loss of ACSL4 leads to macrophage death and converts STING into a driver of IL-1 cytokine release. In a process that has been referred to as transcellular metabolism, TAMs may incorporate AA that has been released by tumor cells, activating signaling pathways similar to those induced by MIF depletion or treatment with nutlin-3a

Implementing single-cell transcriptomics has revealed a level of TAM diversity that cannot be replicated by the traditional activation of macrophages [4]. In this study, which follows a reverse translational approach to clarify mechanisms underlying scRNA-seq-defined TAM subsets [2, 3], we show that accumulation and mitochondrial translocation of p53 in macrophages undergoing M2 differentiation creates a TAM-like phenotype highly reminiscent of IL-1ß^+^ TAMs and IL4I1^+^ TAMs, which have been found in most if not all human tumors [2–4]. A strong feature of the gene expression profile of these TAM-like macrophages was the activation of two key enzymes involved in the metabolism of AA, COX-2 and ACSL4 (Fig. 8). COX-2 produces PGE_2_, which signals via EP2/EP4 receptors to raise cyclic AMP, while ACSL4 converts AA to AA-CoA, enabling Ca^2^⁺ signaling [12] or entry into FA metabolism [9].

In IL-1β⁺ macrophages, NR4A1 drives a distinct gene program induced by the combined action of TNF and PGE₂, known as TP gene signature [2, 22], which, of course, closely resembles the TPP signature [46]. In accordance with the strong activation of TNF and COX-2 in the TAM-like macrophages of the present study, their gene signature resembled the gene expression profiles of TP [22] and TPP macrophages [46], suggesting that NR4A1 also contributes to the gene signature of TAM-like macrophages. In line with this consideration, we found that disruption of MIF signaling not only de-repressed p53 but also NR4A1 and that agonist-induced NR4A1 activation largely recapitulated the TAM-like gene expression profile induced by MIF depletion or nutlin-3a treatment. Strikingly, *IL1B* was the second most strongly activated gene in all three treatment groups (anti-MIF, nutlin-3a, NR4A1 agonist DIM-C-pPhOCH_3_). While our findings implicate NR4A1 in the development of TAM-like macrophages, the much lower degree of gene activation induced by the NR4A1 agonist suggested that NR4A1 (also known as TR3) is not the primary driver but rather facilitates the p53 response [80].

The role of COX-2 in driving PGE_2_ synthesis in TAMs, particularly IL-1ß^+^ TAMs, is well-established [2, 4, 22], but the role of ACSL4 is less clear. Although ACSL4 has been detected in TAMs, it has mostly been studied from the perspective of ferroptosis [81]. However, in MIF-depleted or nutlin-3a-treated human TAM-like macrophages, ACSL4 promoted macrophage survival and prevented IL-1ß release—despite high levels of *IL1B* mRNA—by upregulating CD38 and driving CD38-dependent IL-10 production (Fig. 8). ACSL4 mediated this protective effect by regulating STING. The fact that STING, particularly its receptor function, was also required for CD38 upregulation suggested that the role of ACSL4 is to maintain STING’s homeostatic function [16]. Loss of ACSL4, however, not only caused massive macrophage death, which was difficult to classify, but also converted anti-inflammatory STING into a driver of IL-1 cytokine release. Our observation that p53 engages STING in human macrophages is consistent with previous reports that wild-type p53 can open the mPTP [63] and activate the cGAS-STING pathway in murine models and cell lines [54]. Taken together, co-activation of NR4A1 and p53 gives rise to TAM-like macrophages, which engage an ACSL4/STING-CD38 axis to survive and fuel pathogenic inflammation.

ACSL4 and STING are primarily ER-associated proteins that can physically interact [82]. Under certain conditions, they may also co-localize at mitochondria-associated membranes (MAMs), which link the ER and mitochondria. MAMs serve as metabolically active hubs that integrate calcium, lipid, metabolic, and inflammatory signals [83]. Although our data indicate that ACSL4 regulates STING, the underlying mechanism remains unclear. One possibility involves phosphatidylinositol 4-phosphate (PI4P), a phospholipid implicated in STING activation [16]. Because phosphatidylinositols often contain AA at the sn-2 position, their synthesis depends on AA-CoA generated by ACSL4. In addition, while STING is not intrinsically Ca^2^⁺-dependent, its activity may be influenced by local Ca^2^⁺ dynamics at MAMs. ACSL4-generated AA-CoA may activate Ca^2^⁺ channels, particularly AA-regulated channels (ARCs) [12] by mimicking endogenous ligands [10]. More speculatively, AA-CoA may also influence STING proton channel activity through a similar mechanism.

Beyond MIF depletion or nutlin-3a treatment, exposing macrophages to unesterified AA produced a similar response involving NR4A1, mitochondrial p53, ACSL4/STING, and CD38. Notably, CD38 appeared to maintain its own expression, generating a feed-forward loop.

FA challenge, as we performed it, is used not only to study lipid metabolism, but also to model transcellular biosynthesis. In this process, different cell types cooperate to produce lipid mediators, such as AA-derived eicosanoids, including PGE_2_. Transcellular AA release and metabolism may also occur within tumors [74]. In line with this concept, AA released from tumor or stromal cells may be taken up by monocytes or macrophages and converted into PGE₂, which—via NR4A1—promotes the development of IL-1β⁺ and possibly also IL4I1⁺ TAMs (Fig. 8). Consistently, NR4A1 inactivation blocked nutlin-3a- and AA-induced upregulation of CD38, a hallmark of IL4I1⁺ macrophages [3], suggesting that NR4A1 may indeed also be involved in the development of IL4I1^+^ TAMs. Although MIF is required for proper M2 macrophage differentiation, transcellular AA metabolism appears to be a more likely mechanism for the development of TAMs in the TME [74]. Interestingly, IL-6 and CXCL8, hallmark senescence-associated cytokines [47], were the only cytokines detected in AA-challenged macrophages, suggesting that AA induces senescence [37].

We used multiple criteria to detect senescence in MIF-depleted p53^+^ TAM-like macrophages. The accumulation of p53, a canonical senescence inducer, was paired with ETS1 upregulation and CDK4 downregulation [48, 49], indicating p53-driven cell cycle arrest. These macrophages also displayed transcriptional, secretory, and surface features consistent with senescence [37–39]. Cell death occurred in p53^high^ TAM-like macrophages but not p53^low^ controls after cyclophilin D-mediated mPTP inhibition, supporting senescence [13]. Consistently, cGAS-STING signaling was strongly activated in TAM-like macrophages, aligning with p53-driven mPTP opening [63] and STING activation [54] linked to senescence and aging-related inflammation [14, 55]. Finally, FASN, a key enzyme in lipogenesis needed during senescence induction [8], was essential for generating the TAM-like macrophage secretome.

Our attempts at siRNA- or CRISPR/Cas9-mediated gene knockdown were unsuccessful. While this is generally difficult in primary macrophages due to strong innate immune responses and high endocytic activity, we suspect that p53-driven upregulation of DNA- and RNA-sensing, an apparent feature of senescence [55, 60], further contributed to this limitation. The THP-1 monocytic cell line, often used as a substitute for primary macrophages in knockdown studies, was not suitable here, as it may lack proper p53 activity due to a significant coding sequence deletion [84].

To further probe the role of ACSL4, which was the focus of our study, we used the inhibitor PRGL493, which mimics ACSL4 gene silencing [28]. We also leveraged known off-target effects of ABE and RSG on ACSL4 [72]. Unlike PRGL493, which blocks enzymatic activity [67], ABE also inhibits ACSL4 expression [72]. Our finding that ABE reshapes the TAM surface and secretory phenotype via off-target ACSL4 suppression was notable. The shift towards a more inflammatory state was reflected by increased TNF secretion and reduced TNFR II release. This was intriguing, given that the shedding of the ectodomains of both proteins is mediated by ADAM17 [85]. One of the most notable impacts that ABE had on the TAM secretory phenotype was the suppression of CXCL10, a chemokine that has been associated with oncogenic signaling [73].

In summary, we used a reverse translational approach to elucidate mechanisms potentially underlying scRNA-seq-defined TAM phenotypes. Our findings also help to explain how ABE triggers inflammatory responses in cancer patients [29–31]. ABE has already been tested in combination with PD-1/PD-L1 blockade in clinical studies [86]. Our results further suggest that combining ABE with CD38 inhibition could be a promising strategy.

## Materials and methods

### Reagents and antibodies

The sources of chemicals, recombinant proteins and antibodies used in this study are detailed in Table S1 provided in the Supplementary Material 1.

### Monocyte isolation, macrophage differentiation and treatment

Buffy coats were obtained from the Central Institute for Blood Transfusion and Immunology, University Hospital Innsbruck. To ensure donor anonymity and comply with ethical requirements, buffy coats were provided in a fully anonymized manner. Consequently, no information regarding donor age, sex, gender, or body weight was available to the investigators.

Peripheral blood mononuclear cells (PBMCs) were isolated from buffy coats by density gradient centrifugation using Lymphoprep (Stem Cell Technologies, Vancouver, Canada). Monocytes were then purified from PBMCs by positive selection using CD14 MicroBeads (human; 130–050–201, Miltenyi Biotec) and LS Columns (130–042–401, Miltenyi Biotec), achieving a purity of > 97%. The CD14-negative fraction, which contains T cells and natural killer cells, was used as the responder population in experiments analyzing the immunosuppressive function of macrophages.

Freshly isolated monocytes (1 × 10^6^/·ml^−1^) were cultured in the presence of M-CSF (50 ng/mL) to induce differentiation into M2 macrophages. On day 2, an equal volume of fresh medium containing M-CSF (20 ng·ml^−1^) was added, and on day 5, half of the medium was replaced with fresh M-CSF-supplemented medium (20 ng·ml^−1^).

Fully differentiated M2 macrophages were harvested on day 6, washed, and seeded at 1.5 × 10^5^ cells per well in 300 µL RPMI 1640 medium (PAN-Biotech, Aidenbach, Germany) supplemented with 5% FBS (HyClone, Logan, UT, USA), 1% GlutaMAX (100 ×; Gibco/Thermo Fisher Scientific, Waltham, MA, USA), 10 mM HEPES, 1 mM sodium pyruvate (both PAN-Biotech), 1% non-essential amino acids (NEAA; 100 ×; Gibco), and 1% penicillin/streptomycin (100 U/mL penicillin and 100 µg/mL streptomycin; Gibco) in 48-well plates (Corning Costar, Corning, NY, USA). Following a 2-h equilibration period, cells were treated in duplicate with MIF-neutralizing antibodies (5 µg·ml^−1^) or nutlin-3a (20 µM), in the presence or absence of antagonists/inhibitors, recombinant cytokines, and/or additional neutralizing antibodies, and incubated for 24 h at 37 °C in a humidified atmosphere containing 5% CO2. Subsequently, culture supernatants were collected and stored at −80 °C until further analysis.

To test the effects of exogenous free, unesterified AA, macrophages were treated with a BSA-AA complex composed of AA and FA-free bovine serum albumin (BSA) at a 6:1 molar ratio of AA:BSA (10–30 µM for 24 h; Cayman Chemical).

### Flow cytometry

Cell surface and intracellular antigens were stained with fluorochrome-conjugated monoclonal antibodies (mouse or rat). Corresponding isotype controls were used at the same concentration to determine non-specific background staining. Cells were harvested, washed, and stained with fixable viability dye eFluor 780 (eBioscience/Thermo Fisher Scientific) to exclude dead cells. After washing, cells were incubated with antibodies for 30 min at 4 °C in the dark in PBS (Lonza, Basel, Switzerland) containing 0.5% FBS (HyClone) and 50 µg·ml^−1^ human IgG (Octagam; Octapharma, Lachen, Switzerland) to prevent non-specific Fc receptor binding. Intracellular antigens were stained using the Transcription Factor Staining Buffer Set (130–122–981, Miltenyi Biotec) according to the manufacturer's protocol.

Flow cytometric data were acquired on a FACSCanto II flow cytometer and analyzed using FACS Diva v6.1.2 (RRID:SCR_001456) and FlowJo v7.2.5 software (RRID:SCR_008520) (BD Biosciences). Dead cells were excluded, and doublet discrimination was applied during data analysis.

### Transcriptome analysis

Gene expression was examined using the NanoString’s nCounter analysis system (RRID:SCR_021712). Total RNA was isolated from fully differentiated M2 macrophages treated for 6 h with MIF-neutralizing antibodies (5 µg·ml^−1^) or nutlin-3a (20 µM). RNA was pooled from three different donors. Of each sample, 50 ng of total RNA was used for hybridization reaction with the nCounter Host Response Panel Kit (human; NanoString Technologies, Seattle, WA USA) as described previously [32]. Sample processing was performed at the Core Facility Molecular Biology, Center for Medical Research, Medical University of Graz, Austria. The NanoString platform has been shown to be highly sensitive, reproducible, and technically robust, with results comparable to those obtained using other gene expression profiling technologies (https://www.nanostring.com/scientific-content/publications).

Raw data preprocessing and normalization were performed as previously described [32]. A gene expression threshold of 60 (copy number) was established using negative control probes. Differential expression analysis was conducted using ordinary one-way ANOVA, with genes considered significant at *p* < 0.05 and an absolute FC ≥ 1.5.

### Secretome analysis

Fully differentiated M2 macrophages from three independent donors were treated for 24 h with MIF-neutralizing antibodies (5 µg·ml^−1^) or nutlin-3a (20 µM). CCL20 levels in individual donor supernatants were measured as an internal control to verify successful stimulation. Pooled supernatants were then submitted to a commercial service provider (RayBiotech, Peachtree Corners, GA, USA), quality-controlled, and analyzed in quadruplicate using the Quantibody Human Cytokine Array Q440 (QAH-CAA-440–1; RayBiotech), a quantitative multiplex ELISA array (Human Cytokine Array Q440, RayBiotech).

### Measurements of SASP components

CCL20 (MIP-3α) in cell culture supernatants was quantified by ELISA using the Human CCL20/MIP-3α DuoSet ELISA (R&D Systems). MIF concentrations were measured using the Human MIF ELISA Kit (RayBiotech). Absorbance was read using an Elx800 universal microplate reader (BioTek Instruments/Agilent, Winooski, VT, USA), and data were analyzed with Gen5 v3.09 software (BioTek Instruments/Agilent). Additional SASP factors, including IL-1α, IL-1β, IL-2, IL-6, IL-8, IL-10, VEGF, and TNF-α, were measured in cell culture supernatants using human CBA Flex Sets. Samples were acquired on a FACSCanto II flow cytometer and analyzed using FCAP Array v1.0.1 software (BD Biosciences), according to the manufacturer’s instructions.

### Cell death assessment

Macrophage cultures were examined after 24 h of treatment and documented using an Olympus CK2 microscope with a ProgRes CT3 digital camera and ProgRes CapturePro 2.5 software (Jenoptik). Dead cells were quantified by flow cytometry using fixable viability dye eFluor 780 (eBioscience/Thermo Fisher Scientific).

### Data and statistical analysis

Data are presented as mean ± SD. Sample sizes and numbers of replicates are indicated in the figure legends. Statistical analyses were performed using GraphPad Prism 11.0.0 (RRID:SCR_002798). Differences between two groups were assessed using an unpaired two-tailed *t* test with Welch’s correction. For multiple group comparisons, ordinary one-way ANOVA followed by Šidák’s post hoc test was performed. Statistical significance was defined as *p* < 0.05. Significance levels are denoted as * *p* < 0.05, ** *p* < 0.01, *** *p* < 0.001, and **** *p* < 0.0001. The complete dataset, including all values, *p* values and details of the statistical methods used, is provided in the Supplementary Material 2–3.

## Supplementary Information

Supplementary Material 1. 

Supplementary Material 2. 

Supplementary Material 3.

## Data Availability

All source data including NanoString-based gene expression data and RayBio-based secretome data are available with this paper (Supplementary Material 2–3). Other data are available from the corresponding author upon reasonable request. All materials used are commercially available.
