# Supplementary material for: Disruption of macrophage migration inhibitory factor signaling induces major tumor-associated macrophage phenotypes in human M2 macrophages
Source: Mol Biomed. 2026 Jul 17;7:113. doi: 10.1186/s43556-026-00515-3 (PMC13379542; doi:10.1186/s43556-026-00515-3)
Supplement: Supplementary file 1 — Supplementary Material 1. [file 43556_2026_515_MOESM1_ESM.pdf]

# Supplementary Material 1

Supplementary Figures 1-14 and Supplementary Table 1

# Disruption of macrophage migration inhibitory factor signaling induces major tumor-associated macrophage phenotypes in human M2 macrophages

Dominik Klaver[1*], Hubert Gander[1*], Beatrice Frena[1], Michael Martin[2], Marco Amato[3], Julia Richter[1], Renate Pichler[4], Martin Thurnher[1]

[1]Immunotherapy Unit, Department of Urology, Medical University of Innsbruck, Innsbruck, Austria;

[2]Avidicure B.V., Oegstgeest, The Netherlands;

[3]Central Institute for Blood Transfusion & Department of Immunology (ZIB), Tirol Kliniken GmbH, Innsbruck, Austria

[4]Department of Urology, Medical University of Innsbruck, Innsbruck, Austria

*Authors contributed equally

## Table S1 Reagents and antibodies

| Chemicals, recombinant proteins | Source | Cat # | Concentration | |
|---|---|---|---|---|
| nutlin-3a | Sigma Aldrich | SML0580 | 20 µM | |
| mavorixafor | MedChemExpress | AMD 070 | 10 µM | |
| pifithrin-α | Tocris | 1267 | 25 µM | |
| pifithrin-µ | MedChemExpress | HY-10940 | 10 µM | |
| H-151 | MedChemExpress | HY-112693 | 10 µM | |
| C53 | Tocris | 7741 | 10 µM | |
| cyclosporine A | MedChemExpress | HY-B0579 | 10 µM | |
| NIM811 | MedChemExpress | HY-P0025 | 10 µM | |
| PRGL493 | MedChemExpress | HY-139180 | 10-40 µM | |
| 78c | MedChemExpress | HY-123999 | 2-10 µM | |
| YVAD | Enzo Life Sciences | ALX-260-016 | 50 µM | |
| abemaciclib (ABE) | MedChemExpress | HY-16297A | 2-10 µM | |
| 4-IPP | MedChemExpress | HY-110063 | 20 µM | |
| deferoxamine mesylate (DFO) | Sigma Aldrich | D9533 | 250 µM | |
| TC14012 | MedChemExpress | HY-P1102 | 10 µM | |
| epacadostat | MedChemExpress | HY-15689 | 2 and 5 µM | |
| celecoxib | MedChemExpress | HY-14398 | 20 and 40 µM | |
| triacsin C | MedChemExpress | HY-N6707 | 5 µM | |
| DIM-C-pPhOCH$_3$ | Sigma Aldrich | D7946 | 25 µM | |
| DIM-C-pPhOH | Tocris | 6377 | 2-20 µM | |
| rosiglitazone (RSG) | Sigma Aldrich | R-2408 | 50 µM | |
| C75 | Sigma Aldrich | C5490 | 10 µM | |
| etomoxir | Sigma Aldrich | E1905 | 3 µM | |
| TAPI 1 | Tocris | 6162 | 20 µM | |
| arachidonate-BSA | Cayman Chemical | 34931 | 10-30 µM | |
| M-CSF | Miltenyi Biotec | 130-096-401 | 50 $ng \cdot ml^{-1}$ | |
| recombinant human IL-10 | Sigma Aldrich | H7541 | 50 $ng \cdot ml^{-1}$ | |
| recombinant human IL-2 | Miltenyi Biotec | 130-097-743 | 250 U·ml-1 | |
| **Functional antibodies** | **Source** | **Cat #** | **Identifier** | **Concentration** |
| MIF-neutralizing antibody | Merck Millipore | MABF111 | RRID:AB_1842349 | 5 $\mu g \cdot ml^{-1}$ |
| Isotype control antibody | BD Pharmingen | 554721 | RRID:AB_395530 | 5 $\mu g \cdot ml^{-1}$ |
| CD25 blocking antibody | Invitrogen | BMS134 | RRID:AB_10596650 | 10 $\mu g \cdot ml^{-1}$ |
| CD74 blocking milatuzumab | Invitrogen | MA5-41757 | RRID:AB_2910900 | 100 $ng \cdot ml^{-1}$ |
| **Flow cytometry antibodies** | **Source** | **Cat # / fluorochrome** | **Identifier** | |
| anti-human CD25 | BD Biosciences | 341011 / PE | RRID: AB_2783790 | |
| anti-human CD38 | Miltenyi Biotec | 130-113-433 / Vio Bright FITC | RRID:AB_2726165 | |
| anti-human CD40 | Miltenyi Biotec | 130-110-947 / APC | RRID:AB_2658001 | |
| anti-human CD68 | BD Biosciences | 31883 / FITC | RRID:AB_10896283 | |
| anti-human CD74 | BD Biosciences | 743731 / BV421 | RRID:AB_2741705 | |
| anti-human CD127/IL-7R | BD Biosciences | 5567938 / PE | RRID:AB_2296056 | |
| anti-human CD163 | BD Biosciences | 556018 / PE | RRID: AB_396296 | |
| anti-human CD184/CXCR4 | BD Biosciences | 555974 / PE | RRID:AB_396267 | |
| anti-human CD206 | Invitrogen | 17-2069-42 / APC | RRID:AB_2573182 | |
| anti-human CD274/PD-L1 | BD Biosciences | 563738 / BV421 | RRID:AB_2738396 | |
| anti-human p53 | R&D Systems | IC13551A / APC | RRID:AB_2609736 | |
| anti-human ACSL4 | Santa Cruz | sc-365230 / PE | RRID:AB_10843105 | |
| anti-human IDO1 | BD Biosciences | 567867 / PE | RRID:AB_2916772 | |
| anti-human STING/TMEM173 | R&D Systems | IC7169G / AF488 | RRID:AB_10971940 | |
| anti-human NR4A1 | BD Biosciences | 566735 / AF647 | RRID:AB_2869837 | |

**a**

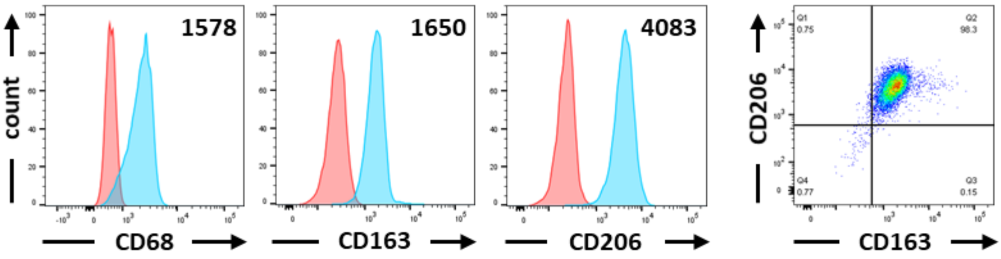


**b**

| Gene | NanoString 1 mRNA copy number | 3 years ↔ Gene | NanoString 2 mRNA copy number |
|---|---|---|---|
| PSAP | 191.956 | PSAP | 58.881 |
| NPC2 | 44.944 | NPC2 | 26.885 |
| LGALS3 | 44.863 | LGALS3 | 26.384 |
| CTSS | 35.932 | CTSS | 19.053 |
| HLA-DRA | 18.707 | HLA-DRA | 14.951 |
| CTSZ | 18.230 | PPIA | 13.081 |
| CCL2 | 17.331 | CTSZ | 12.600 |
| HLA-B | 17.059 | TYROBP | 9.369 |
| CXCL8 | 14.103 | HLA-B | 8.645 |
| TPP1 | 13.315 | CD44 | 8.629 |
| PPIA | 12.461 | CTSL | 8.038 |
| HMOX1 | 12.189 | CD81 | 7.988 |
| CD68 | 11.985 | HMOX1 | 7.852 |
| HLA-E | 11.785 | CD68 | 7.712 |
| HSP90AA1 | 11.022 | DDX5 | 7.467 |
| TXN | 10.886 | CD59 | 7.292 |
| CD163 | 10.795 | TXN | 7.006 |
| CTSA | 10.480 | OAZ1 | 6.794 |
| HSP90B1 | 10.455 | SOD2 | 6.599 |
| CD59 | 10.216 | HLA-DRB | 6.522 |

**c**

| Shared Genes | mRNA copy number |
|---|---|
| PSAP | 58.881 |
| NPC2 | 26.885 |
| LGALS3 | 26.384 |
| CTSS | 19.053 |
| HLA-DRA | 14.951 |
| PPIA | 13.081 |
| CTSZ | 12.600 |
| HLA-B | 8.645 |
| HMOX1 | 7.852 |
| CD68 | 7.712 |
| CD59 | 7.292 |
| TXN | 7.006 |

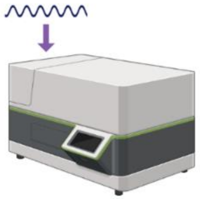


**d**

| Gene symbol | mRNA accession # | Control | |
|---|---|---|---|
| CD2 | NM_001767.3 | 8 | 9 |
| CD3D | NM_000732.4 | 20 | 21 |
| CD3G | NM_000073.2 | 7 | 10 |
| CD6 | NM_001254751.1 | 5 | 4 |
| CD247 | NM_198053.2 | 13 | 13 |
| ICOS | NM_012092.3 | 12 | 10 |
| ITK | NM_005546.3 | 5 | 9 |
| KLRB1 | NM_002258.2 | 20 | 18 |
| TIGIT | NM_173799.3 | 9 | 11 |
| TRAT1 | NM_001317747.1 | 16 | 13 |

**Fig. S1 Human monocyte-derived M-CSF macrophages have an M2-like phenotype with features of senescence**. **a** M2-like phenotype including co-expression of CD206 and CD163 of macrophages differentiating from highly purified monocytes in the presence of M-CSF. Flow cytometry was used to determine surface antigen expression (light blue; isotype controls in red). Numbers are mean fluorescence intensities (MFIs) after subtraction of isotype control MFIs. **b** mRNA copy numbers determined in duplicates using NanoString technology (Host Response Panel; 785 genes) using total RNA isolated from day-6 human M-CSF induced monocyte-derived macrophages (3 donors). The 20 most strongly expressed genes emerging from two independent NanoString analyses carried out at intervals of 3 years (analysis 1: 20.05.2021; analysis 2: 16.04.2024). Values (mRNA copy numbers) were sorted in descending order (median: 483). The top 5 genes were identical. CD68 and CD163 confirm macrophage identity (PMID: 34331874). **c** Overall, 12 of the top 20 genes were identical (60%). According to SeneQuest, which is available at http://Senequest.net, some of these genes including the top 4 (PSAP, NPC2, LGALS3 and CTSS) are related to senescence or its regulation. **d** Lack of T cell specific gene expression (mRNA copy numbers) (Cari et al. 2019; PMID: 31652661) confirms macrophage purity on day 6 (i.e. prior to stimulation) (gene expression threshold: <60). Schematic representation of NanoString analysis: Created in BioRender. Thurnher, M. (2026) https://BioRender.com/9s6x8fi

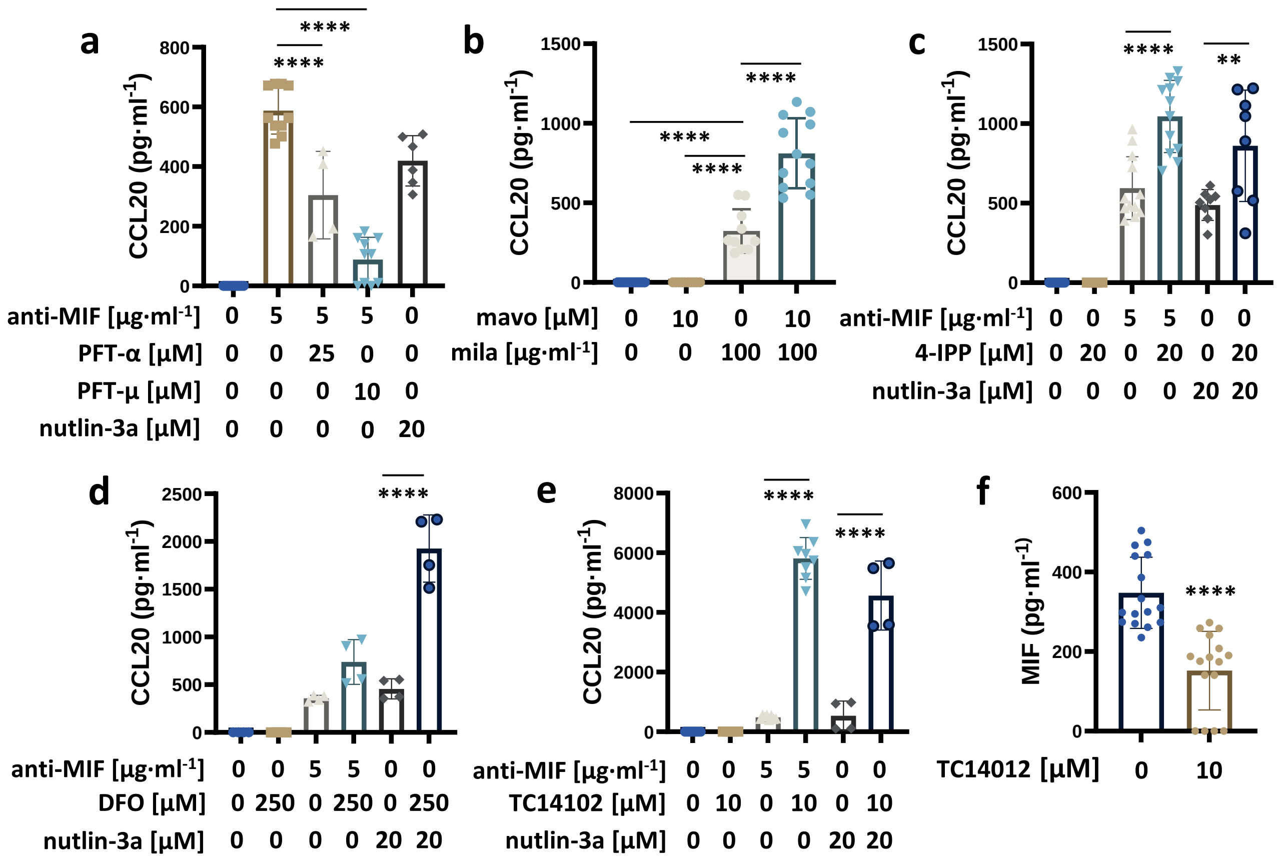

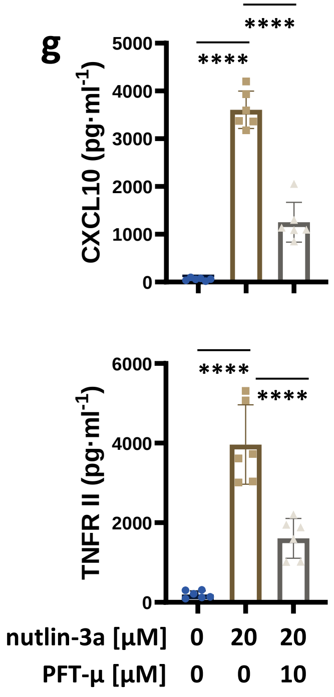


**Fig. S2 MIF depletion induces CCL20 production in human M-CSF macrophages. a** Measurement of CCL20 using ELISA in the cell culture medium of macrophages depleted of macrophage migration-inhibitory factor (MIF) using neutralizing antibody (anti-MIF) or treated with p53 activator nutlin-3a. p53 inhibitors (PFTs) were used to examine nuclear (PFT-α) versus mitochondrial pathways (PFT-µ) (n≥4; one-way ANOVA) **b** measurement of CCL20 upon blockade of MIF receptor components (CXCR4: mavorixafor [mavo]; CD74: milatuzumab [mila]) (n≥10; one-way ANOVA) **c** effect of the intracellular MIF inhibitor 4-iodo-6-phenylpyrimidine (4-IPP) (n≥8; one-way ANOVA) **d** effect of iron chelator deferioxamine (DFO) (n=4; one-way ANOVA) **e** effect of the CXCR7 agonist TC14012 (n=4; one-way ANOVA) **f** Measurement of MIF using ELISA in the cell culture medium of macrophages with or without treatment with the CXCR7 agonist TC14012 (n=16; unpaired *t* test) **g** Measurement of CXCL10 and soluble TNFR II in the cell culture medium of macrophages treated with nutlin-3a: regulation by PFT-µ (n=3; one-way ANOVA).

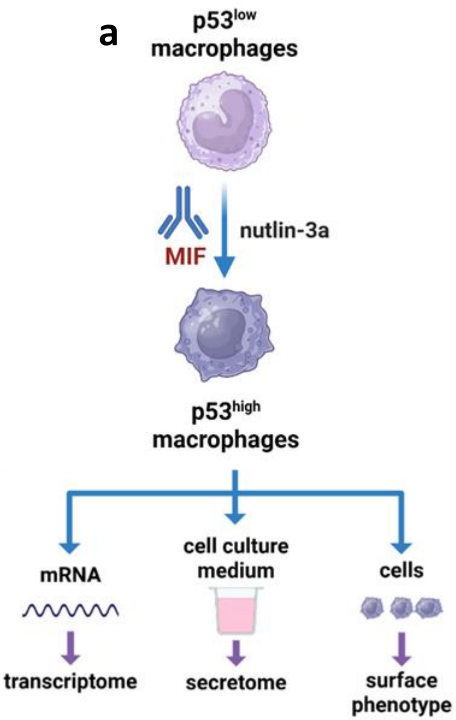


**Fig. S3 Workflow for the analysis of transcriptional, secretory and surface phenotypes. a** Three-pronged approach to investigate reprogramming in macrophages with p53 activation induced either by MIF-depletion or nutlin-3a treatment. Created in BioRender. Thurnher, M. (2026) https://BioRender.com/wizucb4. **b** Day-6 M-CSF (M2-like) macrophages derived from three different donors were subjected to antibody-mediated MIF depletion or nutlin-3a mediated p53 activation. Well plate side view: Created in BioRender. Thurnher, M. (2026) https://BioRender.com/88h6oao. As in our previous studies (PMID: 36107259; PMID: 38472446), CCL20, a dual-function chemokine that is closely related to mitochondrial dysfunction, served as an indicator of successful stimulation. Pooled cell pellets were processed in duplicates for transcriptome analysis (NanoString: 785 genes) and pooled supernatants were used for semi-quantitative secretome analysis (RayBio: 440 soluble components). **c** Global overview showing all genes, which are significantly ($p<0.05$) over- or underexpressed in at least one treatment condition compared to control. Genes were clustered using the ComplexHeatmap R package.

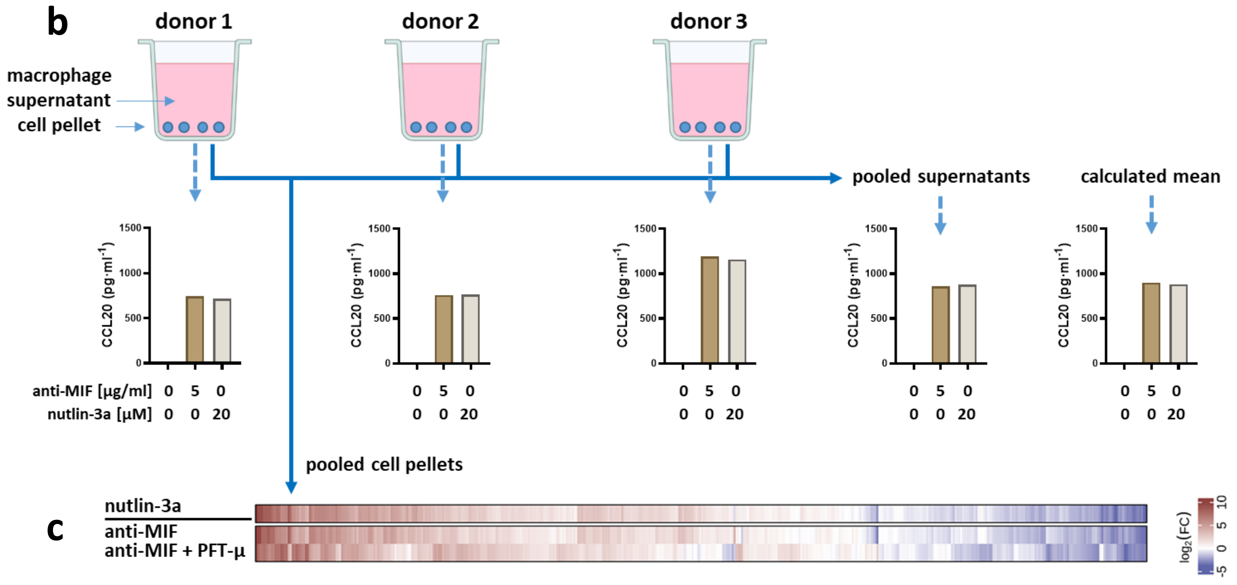

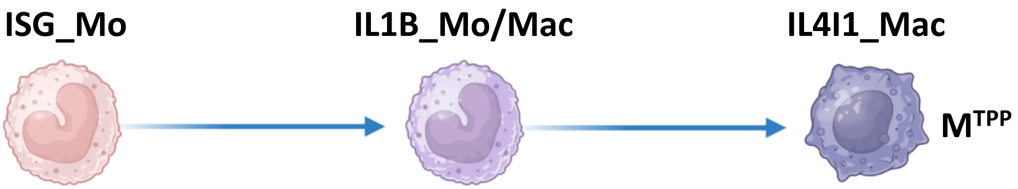


**a**

**ISG_Mo**: Mulder et al. 2021 PMID: 34331874

| Gene | anti-MIF | p value | nutlin-3a | p value |
|---|---|---|---|---|
| IL1RN | 81,16 | 0,0187 | 77,58 | 0,0127 |
| ISG15 | 67,53 | 0,0253 | 175,37 | 0,0172 |
| CXCL10 | 61,06 | 0,0082 | 72,55 | 0,0147 |
| IFIT3 | 31,02 | 0,0158 | 50,18 | 0,0010 |
| TNFSF10 | 25,69 | 0,0069 | 33,55 | 0,0045 |
| CCL8 | 25,17 | 0,0307 | 23,68 | 0,0603 |
| IFIT1 | 21,09 | 0,0053 | 28,12 | 0,0168 |
| IFIT2 | 14,32 | 0,0093 | 33,92 | 0,0059 |
| IRF7 | 10,15 | 0,0174 | 26,96 | 0,0047 |
| TNFSF13B | 6,08 | 0,0156 | 7,89 | 0,0060 |
| STAT1 | 4,63 | 0,0171 | 4,65 | 0,0085 |

**b**

**IL1B_Mac**: Mulder et al. 2021 PMID: 34331874

| Gene | anti-MIF | p value | nutlin-3a | p value |
|---|---|---|---|---|
| IL1B | 969,44 | 0,0177 | 706,82 | 0,0098 |
| CCL20 | 913,71 | 0,0102 | 669,47 | 0,0116 |
| IL1RN | 81,16 | 0,0187 | 77,58 | 0,0127 |
| CCL4/L1/L2 | 55,97 | 0,0311 | 71,68 | 0,0337 |
| CXCL3 | 20,46 | 0,0069 | 12,94 | 0,0082 |
| VEGFA | 9,43 | 0,0387 | 9,91 | 0,0445 |
| CXCL2 | 7,81 | 0,0429 | 10,44 | 0,0394 |
| PLAUR | 4,48 | 0,0795 | 5,97 | 0,0648 |
| CD44 | 3,89 | 0,0852 | 5,40 | 0,0682 |

**c**

**IL-1ß⁺ Mac**: Caronni et al. 2023 PMID: 37914939

| Gene | anti-MIF | p value | nutlin-3a | p value |
|---|---|---|---|---|
| IL1B | 969,44 | 0,0177 | 706,82 | 0,0098 |
| PTGS2 | 345,12 | 0,0021 | 333,05 | 0,0000 |
| CXCL3 | 20,46 | 0,0069 | 12,94 | 0,0082 |
| NLRP3 | 14,71 | 0,0016 | 13,85 | 0,0014 |
| THBS1 | 14,66 | 0,0524 | 17,61 | 0,0385 |
| TNF | 9,47 | 0,0096 | 12,50 | 0,0073 |
| VEGFA | 9,43 | 0,0387 | 9,91 | 0,0445 |
| CXCL2 | 7,81 | 0,0429 | 10,44 | 0,0394 |

**d**

**IL4I1_Mac**: Mulder et al. 2021 PMID: 34331874

| Gene | anti-MIF | p value | nutlin-3a | p value |
|---|---|---|---|---|
| IDO1 | 971,67 | 0,0110 | 1467,26 | 0,0031 |
| LAMP3 | 259,47 | 0,0140 | 348,58 | 0,0102 |
| CD274 | 231,25 | 0,0008 | 329,43 | 0,0033 |
| CXCL11 | 210,34 | 0,0045 | 364,41 | 0,0117 |
| CXCL10 | 61,06 | 0,0082 | 72,55 | 0,0147 |
| CD38 | 53,98 | 0,0015 | 88,14 | 0,0012 |
| CD40 | 29,54 | 0,0144 | 38,05 | 0,0191 |
| CCL8 | 25,17 | 0,0307 | 23,68 | 0,0603 |
| CXCL9 | 8,51 | 0,0322 | 25,97 | 0,0070 |
| STAT1 | 4,63 | 0,0171 | 4,65 | 0,0085 |

**e**

**$M^{TPP}$**: Xue et al. 2014 PMID: 24530056

| Gene | anti-MIF | p value | nutlin-3a | p value |
|---|---|---|---|---|
| CD25 | 342,78 | 0,0037 | 403,91 | 0,0013 |
| PTGS2 | 345,12 | 0,0021 | 333,05 | 0,0000 |
| IDO1 | 971,67 | 0,0110 | 1467,26 | 0,0031 |
| IL1A | 81,92 | 0,0020 | 45,99 | 0,0282 |
| IL10 | 41,83 | 0,0136 | 49,20 | 0,0151 |
| CXCL5 | 19,76 | 0,0226 | 12,75 | 0,0317 |
| CXCR7 | 7,97 | 0,0968 | 3,81 | 0,1045 |

**Fig. S4. Alignment of gene expression data obtained from MIF-depleted or nutlin-3a treated macrophages to previously defined clusters. a-e** Mulder et al. (ISG_Mo; IL1B_Mo/Mac; IL4I1-Mac) (PMID: 34331874) and Caronni et al. (IL-1ß⁺ TAM) (PMID: 37914939) as well as Xue et al. ($M^{TPP}$) (PMID: 24530056). The data illustrate that the phenotypes of these distinct subsets can emerge in a cell-autonomous manner, when macrophages enter a continuous differentiation path (fold changes over control are shown). Schematic representation of macrophage differentiation: Created in BioRender. Thurnher, M. (2026) https://BioRender.com/9tq9b7p

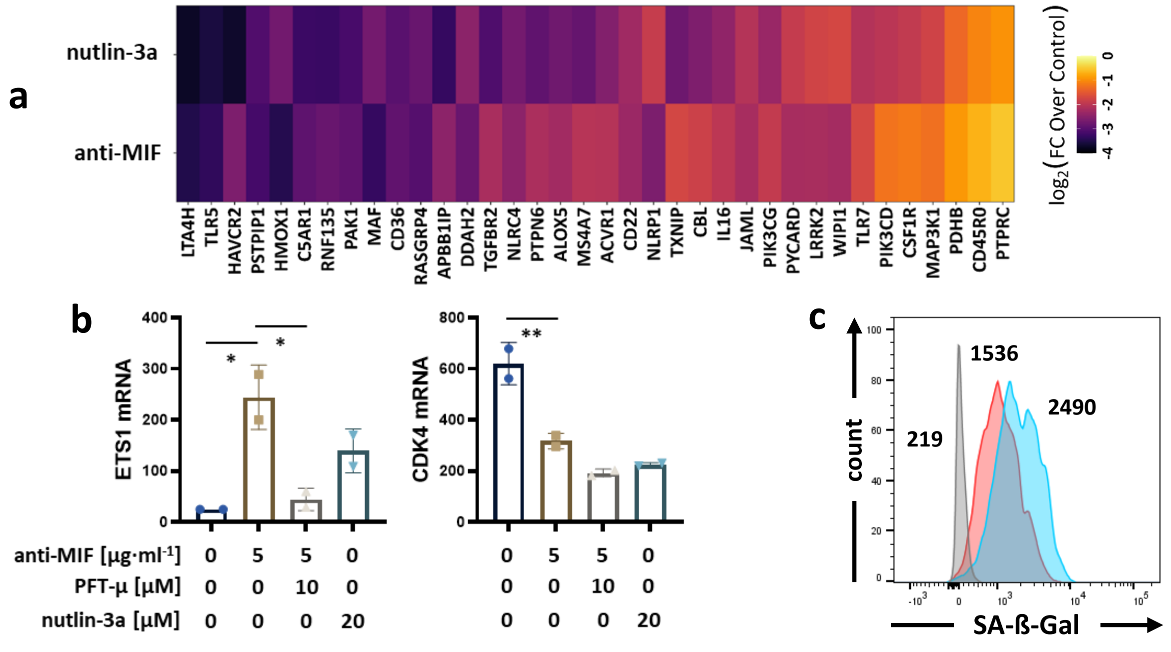


**d**

| Gene | anti-MIF control | p value | anti-MIF + PFT-µ control | p value | nutlin-3a control | p value |
|---|---|---|---|---|---|---|
| IL1A | 81,92 | 0,0020 | 16,15 | 0,0076 | 45,99 | 0,0282 |
| IL1B | 969,44 | 0,0177 | 214,84 | 0,0320 | 706,82 | 0,0098 |
| IL1R1 | 5,56 | 0,0268 | 6,25 | 0,0198 | 2,60 | 0,0841 |
| IL1RAP | 8,24 | 0,0155 | 2,49 | 0,0753 | 8,25 | 0,0330 |
| IL1RN | 81,16 | 0,0187 | 23,79 | 0,0069 | 77,58 | 0,0127 |
| PTPN6 | -4,46 | 0,0181 | -3,60 | 0,0219 | -7,49 | 0,0146 |
| SIGRR | -3,68 | 0,0012 | -1,94 | 0,1084 | -2,95 | 0,0238 |
| THBS1 | 14,66 | 0,0524 | 2,30 | 0,1738 | 17,61 | 0,0385 |

**Fig. S5 Reprogramming of human macrophages in response to MIF depletion or treatment with nutlin-3a. a** Heatmap of the genes most significantly underexpressed ($p<0.05$) in either MIF-depleted or nutlin-3a treated, i.e. p53-activated, human macrophages versus control macrophages as assessed by NanoString-based gene expression analysis using the Host Response Panel. **b** Quantification of ETS1 and CDK4 mRNA (copy numbers) using NanoString technology (Host Response Panel) (n=3; one-way ANOVA). **c** Flow cytometric assessment of SA-ß-Gal in p53-activated (red) versus control macrophages (light blue; unstained control: gray;) (n.s.). **d** Fold change of genes related to IL-1 signaling. The IL-1R ligands, IL1A and IL1B, as well as the IL-1 receptor components IL1R1 and IL1RAP were all upregulated. IL1RN, encoding IL-1R antagonist, was also upregulated. In contrast PTPN6 and SIGRR, both negative regulators of IL-1R signaling (PMID: 18806225 PMID: 12925853), were downregulated. Of note, SHP-1, encoded by PTPN6, can be repressed by p53 (PMID: 19749791). THBS1, encoding thrombospondin-1, has been described as part of the IL-1ß program of macrophages associated with pancreatic cancer (PMID: 26813769). THBS1 has also been implicated in IL-1ß regulation in macrophages (PMID: 26813769).

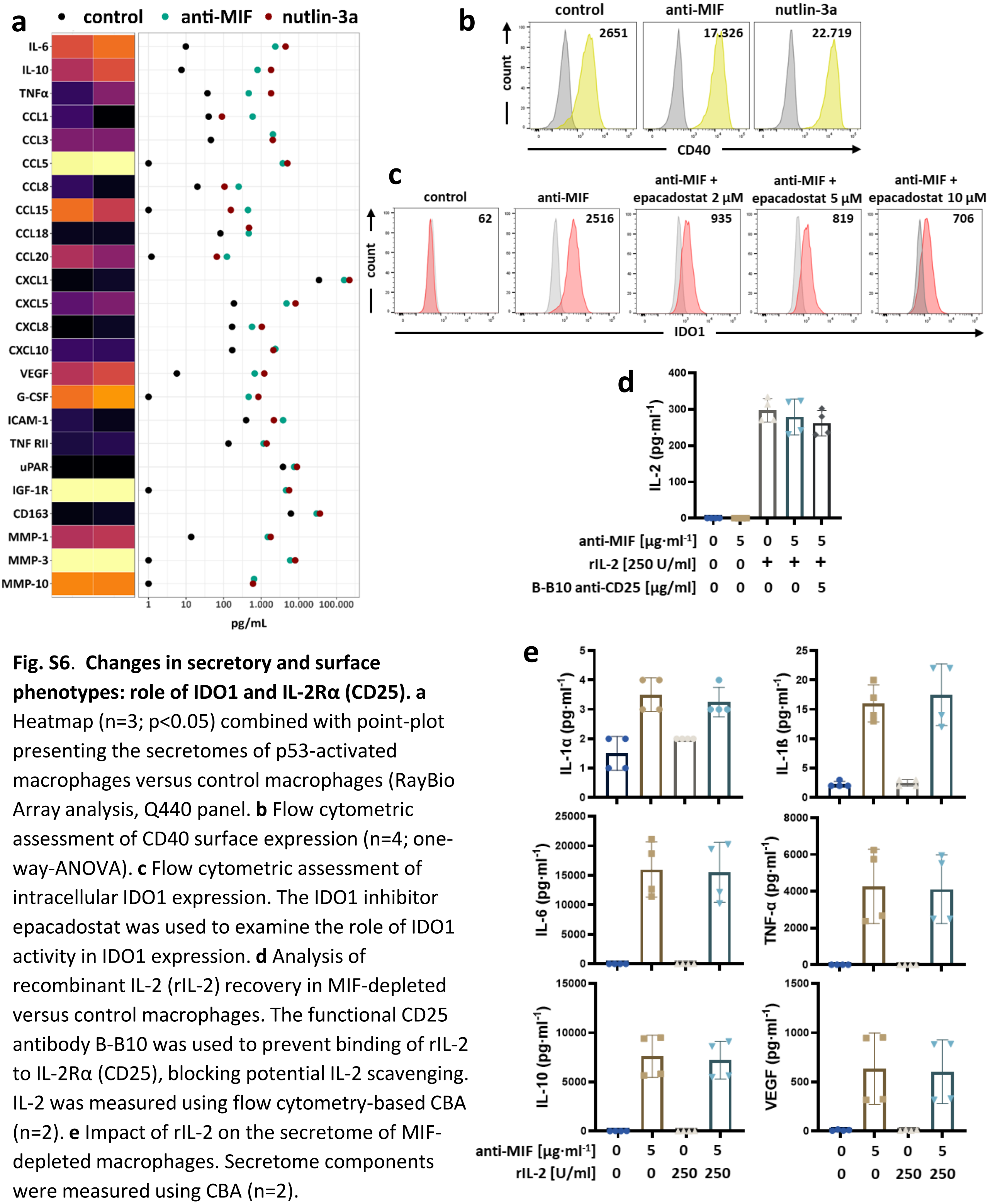


**Fig. S6. Changes in secretory and surface phenotypes: role of IDO1 and IL-2Rα (CD25). a** Heatmap (n=3; p<0.05) combined with point-plot presenting the secretomes of p53-activated macrophages versus control macrophages (RayBio Array analysis, Q440 panel. **b** Flow cytometric assessment of CD40 surface expression (n=4; one-way-ANOVA). **c** Flow cytometric assessment of intracellular IDO1 expression. The IDO1 inhibitor epacadostat was used to examine the role of IDO1 activity in IDO1 expression. **d** Analysis of recombinant IL-2 (rIL-2) recovery in MIF-depleted versus control macrophages. The functional CD25 antibody B-B10 was used to prevent binding of rIL-2 to IL-2Rα (CD25), blocking potential IL-2 scavenging. IL-2 was measured using flow cytometry-based CBA (n=2). **e** Impact of rIL-2 on the secretome of MIF-depleted macrophages. Secretome components were measured using CBA (n=2).

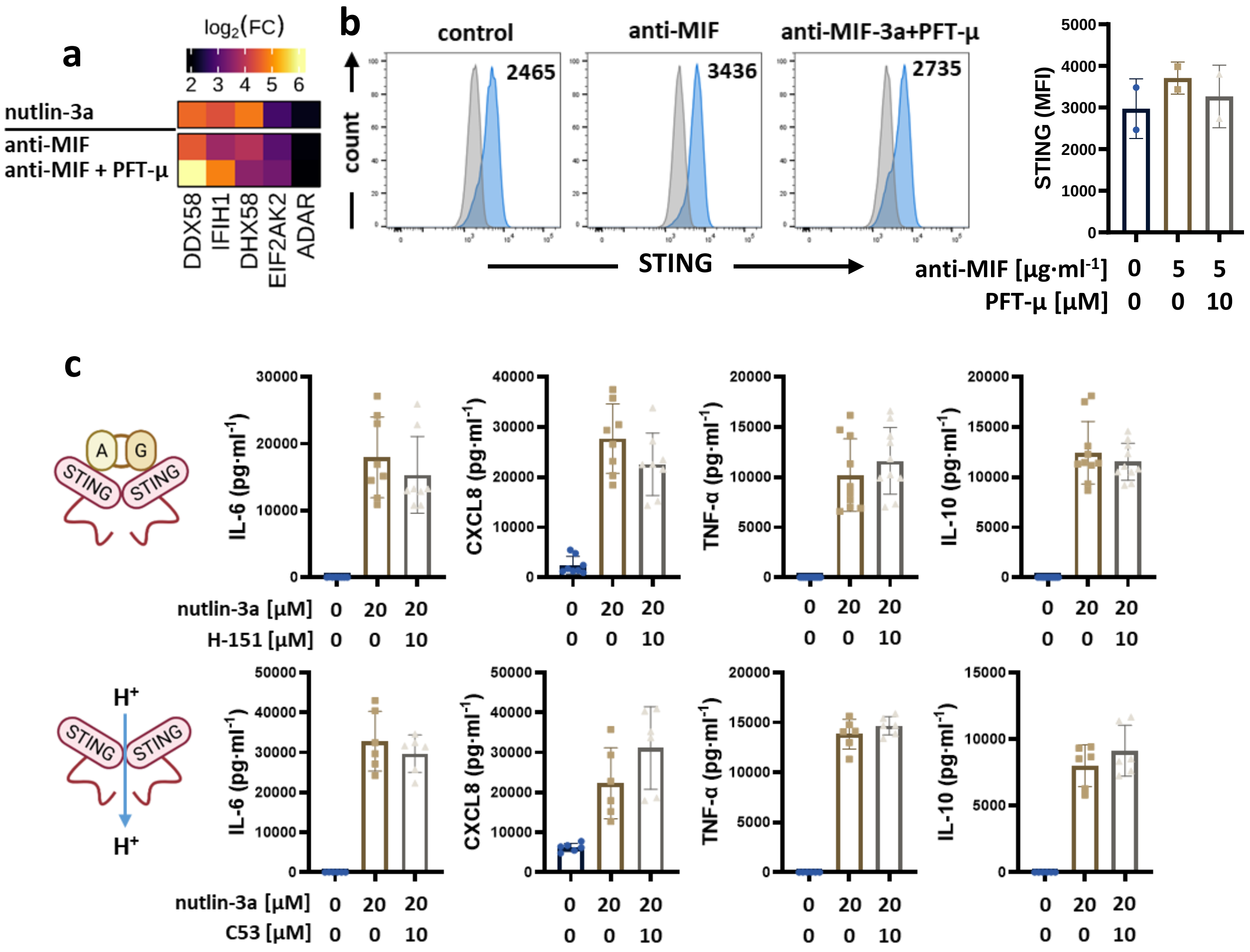


**Fig. S7. Activation of the RNA and DNA-sensing in p53-activated macrophages. a** Heatmap showing upregulation of the main RNA sensors in response to nutlin-3a treatment or MIF depletion (n=3; $p<0.05$). **b** Intracellular flow cytometric assessment of the DNA senor STING in MIF-depleted versus control macrophages, and quantification. **a-b** The role of mitochondrial p53 was examined using PFT-µ. **c** Measurement of SASP components using CBA in the cell culture medium of nutlin-3a treated versus control macrophages. The role of STING was examined using the inhibitors, H-151 (covalent STING antagonist) and C53 (inhibitor of STING's proton channel activity) (n≥3). The induction of all SASP components following nutlin-3a treatment was highly significant ($p < 0.0001$). Changes induced by H-151 or C53 were not significant. Schematic representation of STING's receptor and proton channel function: Created in BioRender. Thurnher, M. (2026) https://BioRender.com/huss17j

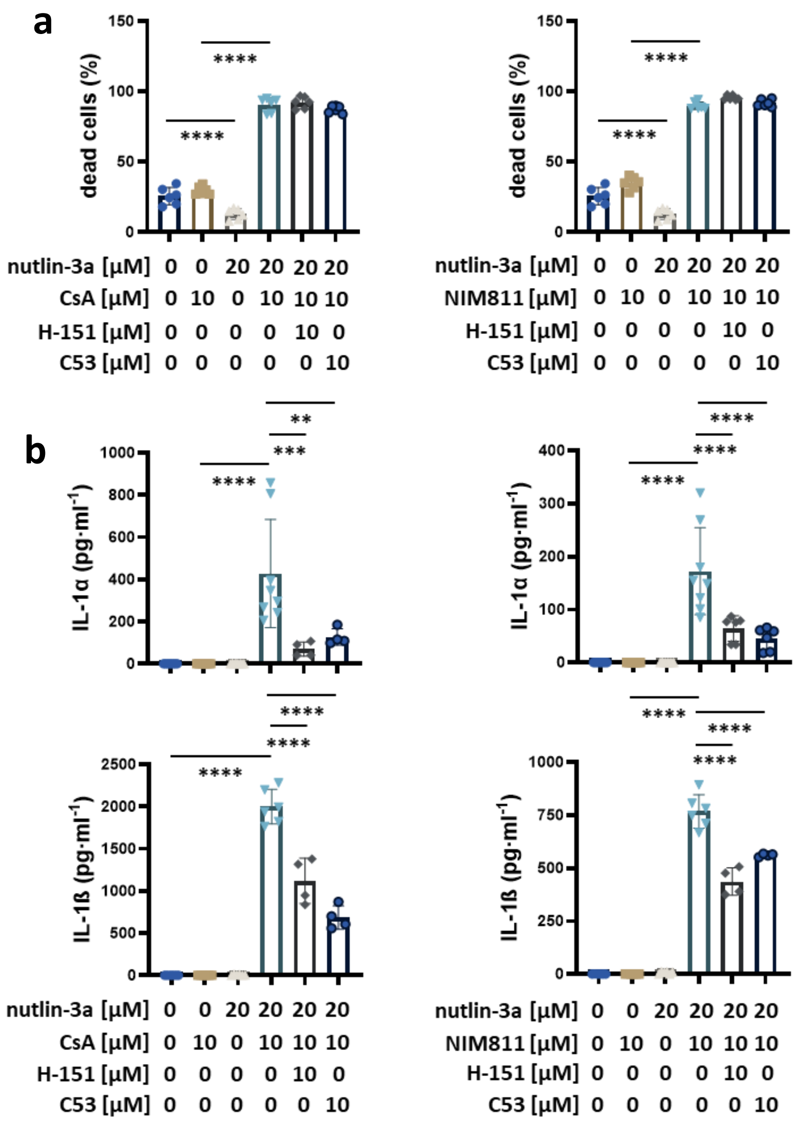


**Fig. S8 p53$^{high}$ TAM-like macrophages are sensitive to cell death induced by inhibitors of the mPTP: cell death is accompanied by STING-driven IL-1 secretion. a** Dead cells were quantified by flow cytometry using fixable viability dye eFluor 780 in cultures of nutlin-3a treated versus control macrophages upon cyclophilin D inhibition using cyclosporin A or its derivative NIM811 lacking inhibitory activity towards calcineurin. The role of STING was examined using the inhibitors H-151 and C53 (n=3; one-way ANOVA) **b** CBA-based assessment of IL-1α and IL-1ß (n≥3: one-way ANOVA).

**a**

| gene | anti-MIF control | p value | anti-MIF+PFT-μ control | p value | nutlin-3a control | p value |
|---|---|---|---|---|---|---|
| PTGS2 | 345,12 | 0,0021 | 75,10 | 0,0001 | 333,05 | 0,0000 |
| PTGER2 | 4,88 | 0,0672 | 4,14 | 0,0650 | 3,89 | 0,0632 |
| PTGER4 | 2,57 | 0,0940 | 1,37 | 0,3812 | 2,63 | 0,1092 |
| TBXAS1 | -2,83 | 0,0153 | -1,55 | 0,0577 | -3,01 | 0,0020 |
| ALOX5 | -4,80 | 0,0011 | -7,61 | 0,0161 | -6,78 | 0,0055 |
| ALOX5AP | -1,34 | 0,0489 | 1,06 | 0,6835 | -1,86 | 0,0468 |
| LTA4H | -11,27 | 0,0469 | -7,43 | 0,0736 | -13,32 | 0,0531 |
| STAT1 | 4,63 | 0,0171 | 6,37 | 0,0293 | 4,65 | 0,0085 |
| STAT2 | 4,35 | 0,0384 | 3,30 | 0,0530 | 7,38 | 0,0271 |
| STAT3 | 3,11 | 0,0145 | 1,68 | 0,0169 | 3,71 | 0,0052 |
| STAT4 | 61,92 | 0,0011 | 15,25 | 0,0047 | 75,28 | 0,0014 |

**b**

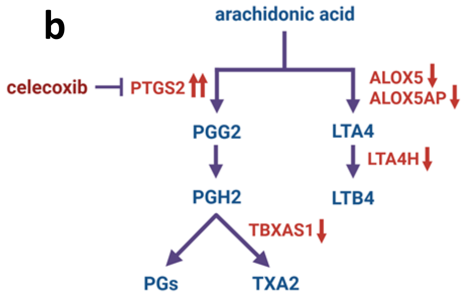


**c**

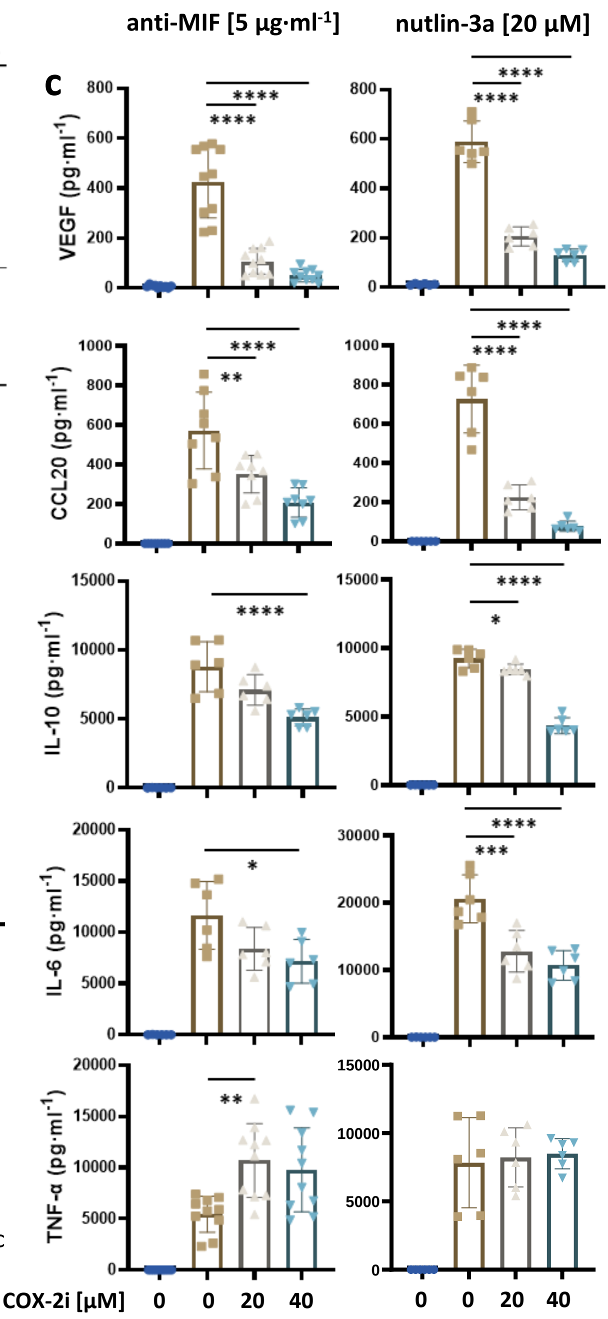


**d**

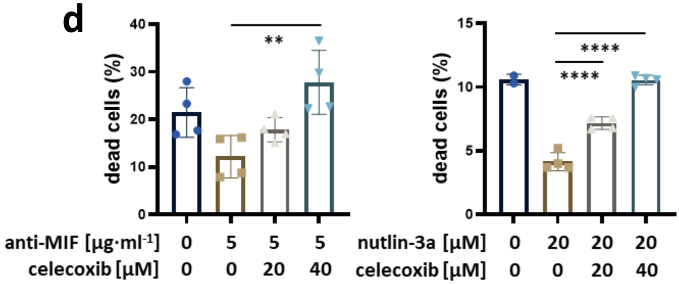


**Fig. S9 Arachidonic acid metabolism in p53-activated macrophages. a** Gene regulation in p53-activated macrophages was assessed by NanoString-based mRNA quantification (FC over control is shown). The role of mitochondrial p53 was examined using PFT-μ. **b** Schematic illustrating channelled flux towards PGs as a result of metabolic reprogramming. Arrows indicate up- or downregulation of genes. Created in BioRender. Thurnher, M. (2026) https://BioRender.com/pa0fdok **c** Measurement of SASP components using CBA or ELISA (CCL20). The role of COX-2 (PTGS2) was examined using COX-2 inhibitor celecoxib (COX-2i). **d** Dead cells were quantified using fixable viability dye eFluor 780. n≥3, one-way ANOVA;

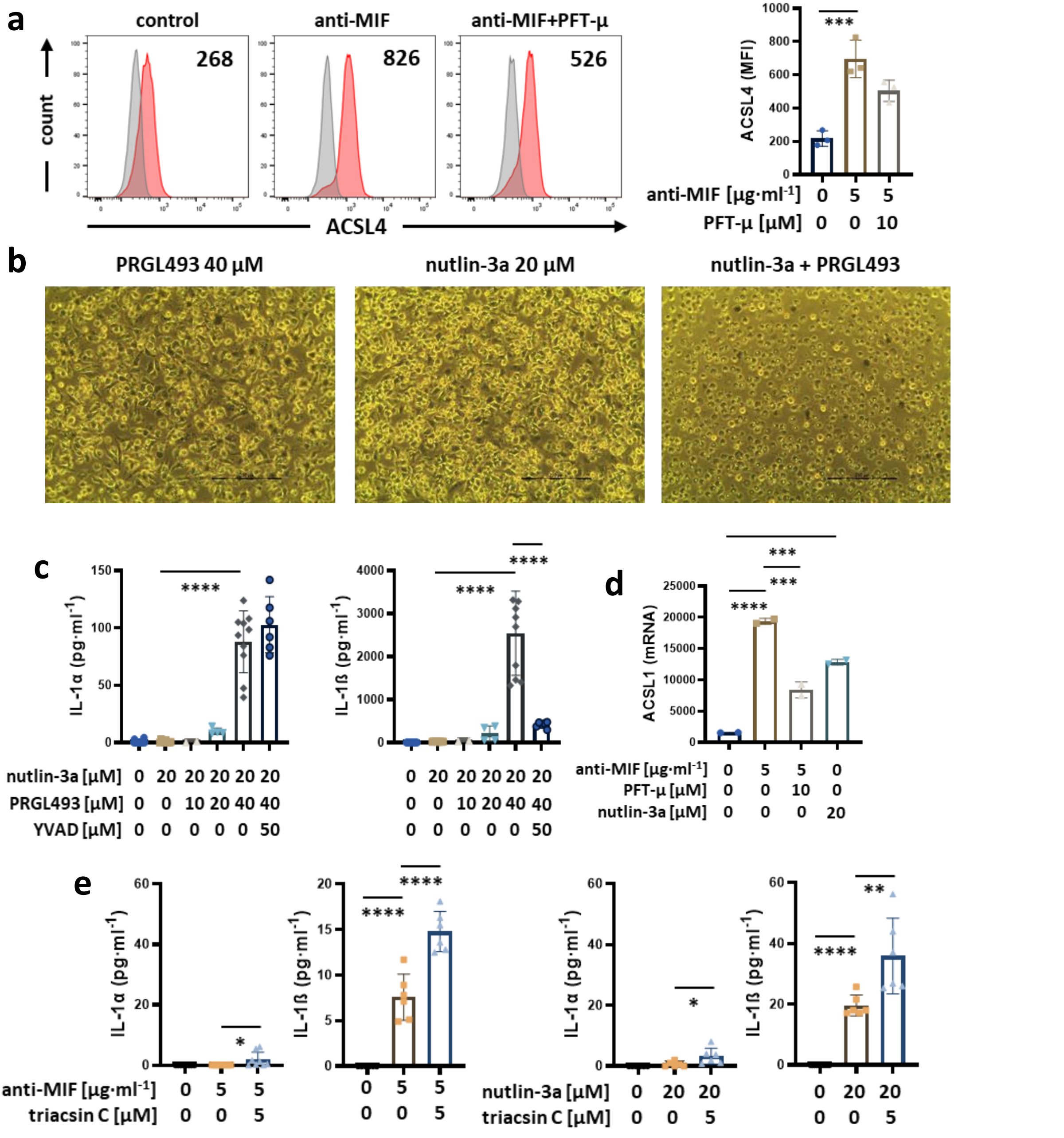


**Fig. S10 ACSL4 protein expression and function in human TAM-like macrophages. a** Flow cytometric assessment of intracellular ACSL4. The role of mitochondrial p53 was examined using PFT-µ. Representative histograms and quantification (n=3; one-way ANOVA. **b** Phase contrast microscopy of macrophages (magnification: 100x). The role of ACSL4 was examined using PRGL493. **c** CBA-based measurement of IL-1α and IL-1ß induced by ACSL4 inhibitor PRGL493 and regulated by caspase-1 inhibitor YVAD (n≥3; one-way ANOVA). **d** Quantification of ACSL1 mRNA copy numbers using NanoString technology (n=3; one-way ANOVA). **e** IL-1 cytokine measurement: the role of ACSL1 was examined using triacsin C (n≥3; one-way ANOVA).

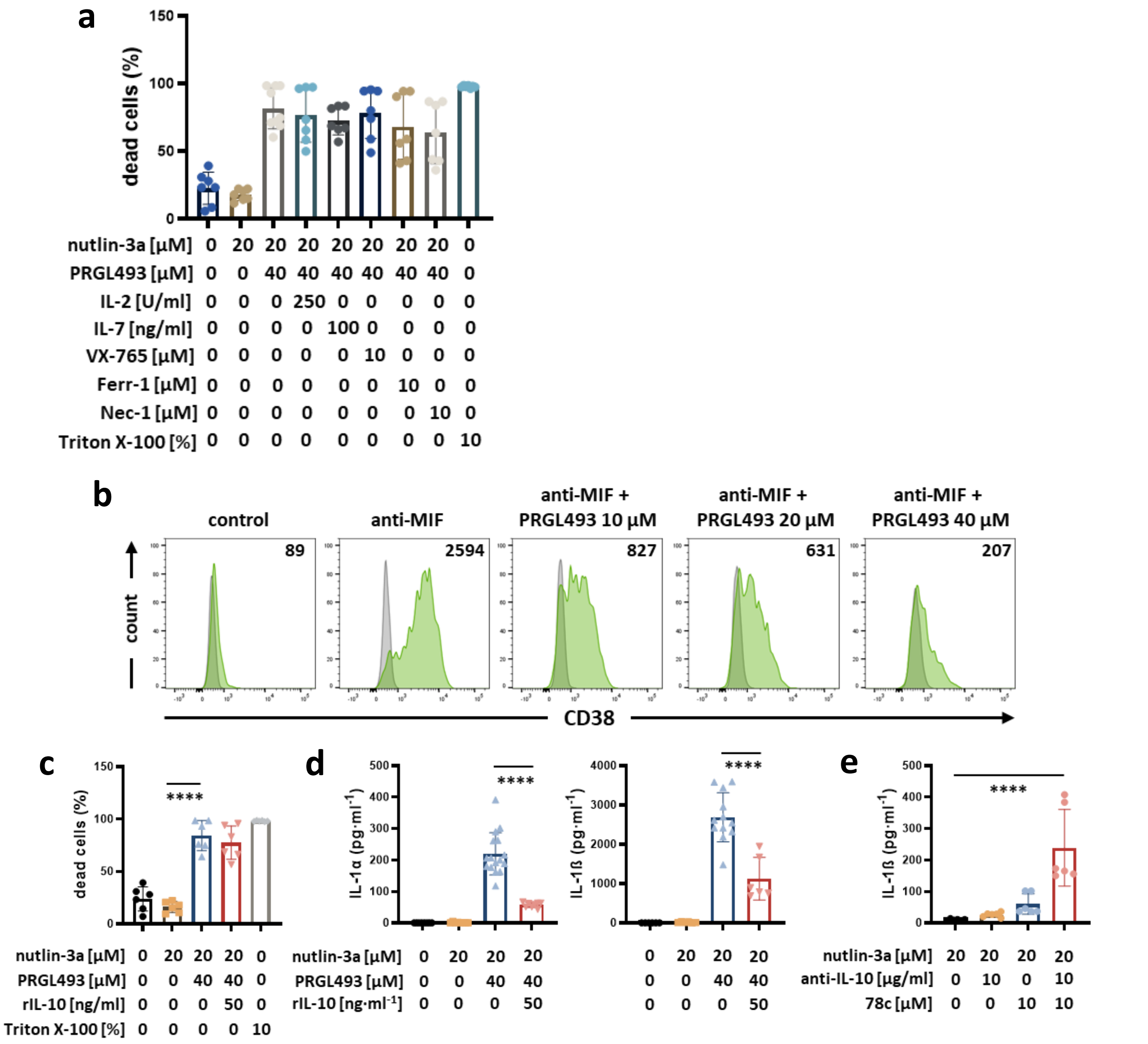


**Fig. S11 ACSL4 ensures macrophage survival and promotes CD38 expression in TAM-like macrophages. a** Cell death was induced by combined treatment of macrophages with nutlin-3a and ACSL4 inhibitor PRGL493. Cytokines as well as other compounds were tested for their ability to prevent cell death. None of the substances tested caused any significant changes. Dead cells were quantified by flow cytometry using fixable viability dye eFluor 780. **b** Flow cytometric assessment of CD38 surface expression. The role of ACSL4 was examined using PRGL493 (n=2; one-way ANOVA). **c** Recombinant IL-10 (rIL-10) was added to compensate for PRGL493-mediated inhibition of endogenous IL-10 and dead cells were quantified (n=3; one-way ANOVA) **d** CBA-based measurement of IL-1α and IL-1ß induced by ACSL4 inhibitor PRGL493 and regulated by rIL-10 (n≥3; one-way ANOVA). **e** CBA-based measurement of IL-1ß induced by IL-10 neutralization and CD38 inhibition (78c) (n=3; one-way ANOVA).

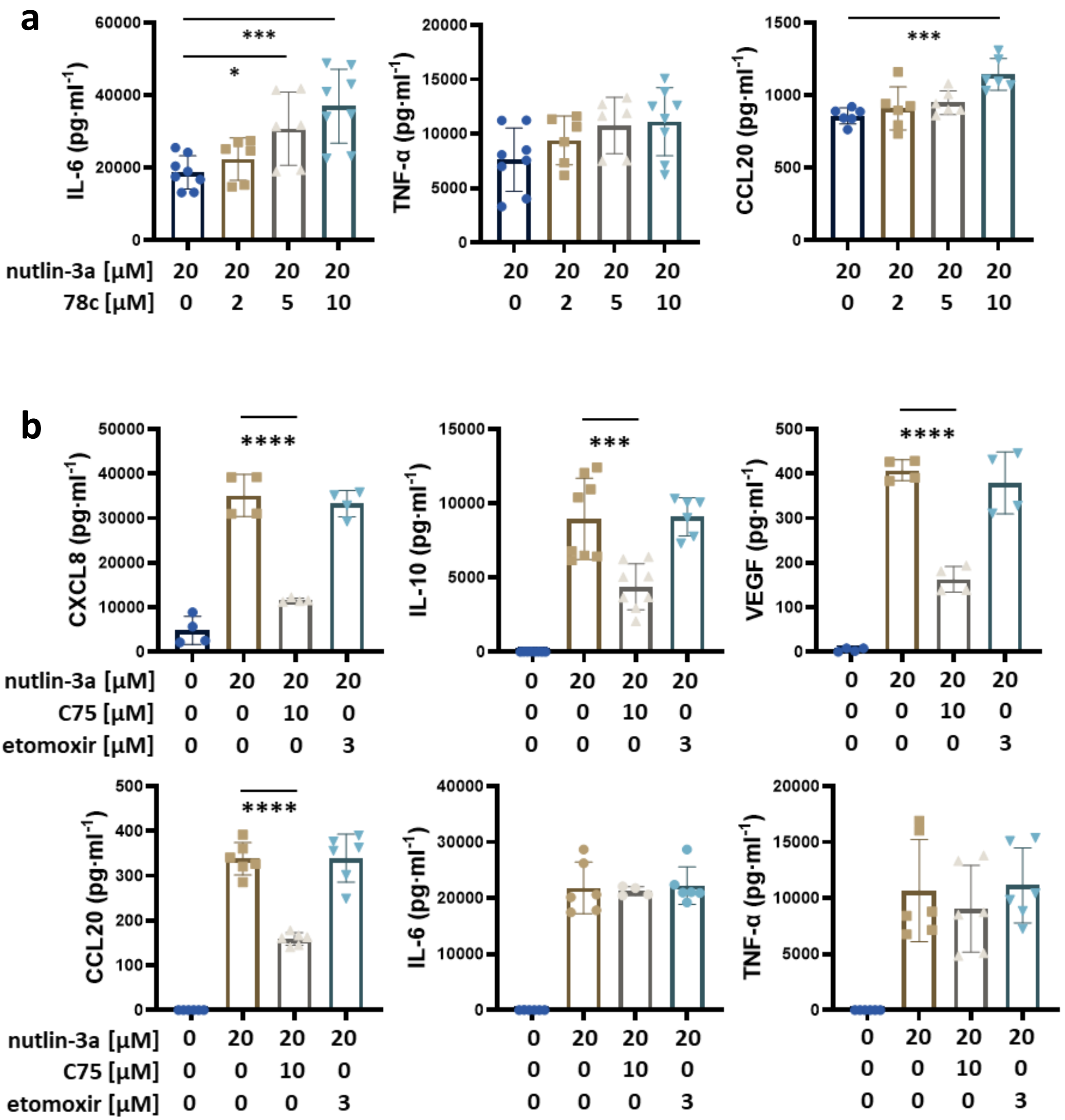


**Fig. S12 Impact of CD38 and FASN inhibition on the secretome of TAM-like macrophages. a** Treatment of TAM-like macrophages with increasing doses of CD38 inhibitor 78c (n≥3; one-way ANOVA). **b** Treatment of TAM-like macrophages with FASN inhibitor C75 or FA oxidation inhibitor etomoxir. Etomoxir was tested at a maximum concentration of 3 µM to avoid off-target effects (PMID: 30043752) (n≥2; one-way ANOVA). **a-b** ELISA-based (CCL20) or CBA-based assessment of SASP components.

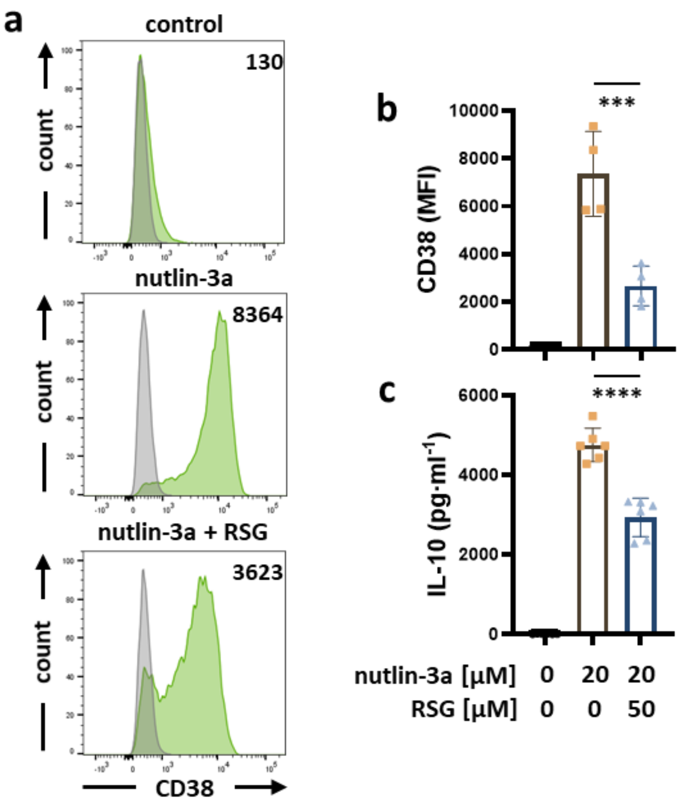


**Fig. S13 Rosiglitazone (RSG) attenuates nutlin-3a induced CD38 upregulation and IL-10 expression.** **a** Flow cytometric analysis of CD38 surface expression on nutlin-3a treated versus control macrophages and regulation by RSG, which emerged alongside abemaciclib as an inhibitor of ACSL4 (PMID: 34510514). **b** Quantification of CD38 expression (n=4; one-way ANOVA) **c** CBA-based measurement of IL-10 released from nutlin-3a treated macrophages and regulated by RSG (n=3; one-way ANOVA).

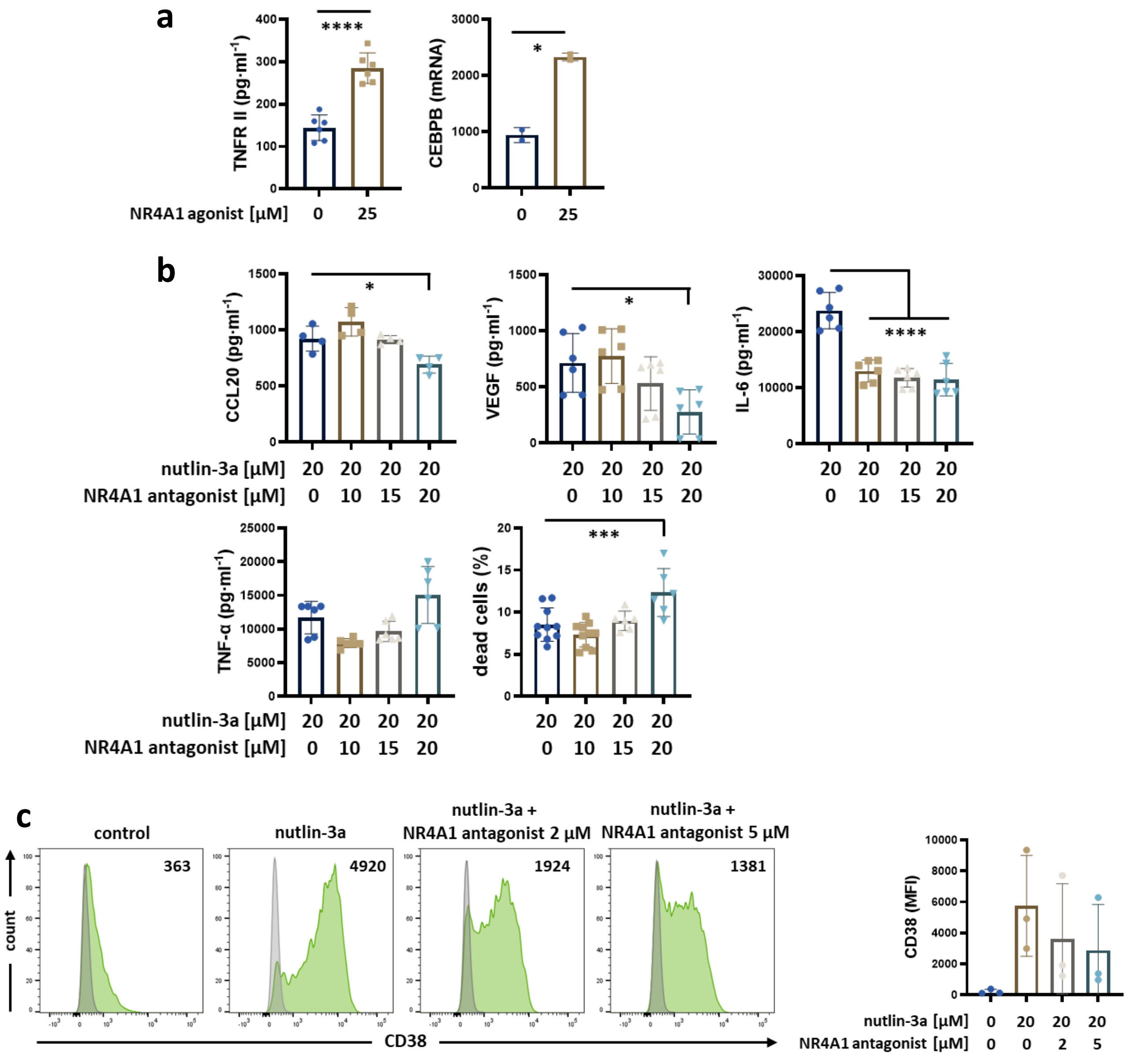


**Fig. S14 Effects of NR4A1 agonism and antagonism in control and TAM-like macrophages. a** Treatment of control macrophages with NR4A1 agonist DIM-C-pPhOC$H_3$: CBA-based measurement of soluble TNFR II and NanoString-based assessment of CEBPB mRNA copy numbers (n=3; unpaired *t* test). **b** Treatment of TAM-like macrophages with increasing doses of NR4A1 antagonist DIM-C-pPhOH and ELISA-based assessment of CCL20 or CBA-based assessment of TNF. Dead cells were quantified by flow cytometry using fixable viability dye eFluor 780 (n≥2). **c** Treatment of TAM-like macrophages with low doses of NR4A1 antagonist and flow cytometric assessment of CD38 surface expression (n=3; one-way ANOVA; not significant).
